# Extracellular Vesicles Mediate Radiation-Induced Systemic Bystander Signals in the Bone Marrow and Spleen

**DOI:** 10.3389/fimmu.2017.00347

**Published:** 2017-03-27

**Authors:** Tünde Szatmári, Dávid Kis, Enikő Noémi Bogdándi, Anett Benedek, Scott Bright, Deborah Bowler, Eszter Persa, Enikő Kis, Andrea Balogh, Lívia N. Naszályi, Munira Kadhim, Géza Sáfrány, Katalin Lumniczky

**Affiliations:** ^1^Division of Radiation Medicine, National Public Health Centre, National Research Directorate for Radiobiology and Radiohygiene, Budapest, Hungary; ^2^Genomic Instability Group, Department of Biological and Medical Sciences, Oxford Brookes University, Oxford, UK; ^3^Research Group for Molecular Biophysics, Hungarian Academy of Sciences, Semmelweis University, Budapest, Hungary

**Keywords:** ionizing radiation, hematopoietic system, microRNA, extracellular vesicles, bystander effects

## Abstract

Radiation-induced bystander effects refer to the induction of biological changes in cells not directly hit by radiation implying that the number of cells affected by radiation is larger than the actual number of irradiated cells. Recent *in vitro* studies suggest the role of extracellular vesicles (EVs) in mediating radiation-induced bystander signals, but *in vivo* investigations are still lacking. Here, we report an *in vivo* study investigating the role of EVs in mediating radiation effects. C57BL/6 mice were total-body irradiated with X-rays (0.1, 0.25, 2 Gy), and 24 h later, EVs were isolated from the bone marrow (BM) and were intravenously injected into unirradiated (so-called bystander) animals. EV-induced systemic effects were compared to radiation effects in the directly irradiated animals. Similar to direct radiation, EVs from irradiated mice induced complex DNA damage in EV-recipient animals, manifested in an increased level of chromosomal aberrations and the activation of the DNA damage response. However, while DNA damage after direct irradiation increased with the dose, EV-induced effects peaked at lower doses. A significantly reduced hematopoietic stem cell pool in the BM as well as CD4^+^ and CD8^+^ lymphocyte pool in the spleen was detected in mice injected with EVs isolated from animals irradiated with 2 Gy. These EV-induced alterations were comparable to changes present in the directly irradiated mice. The pool of TLR4-expressing dendritic cells was different in the directly irradiated mice, where it increased after 2 Gy and in the EV-recipient animals, where it strongly decreased in a dose-independent manner. A panel of eight differentially expressed microRNAs (miRNA) was identified in the EVs originating from both low- and high-dose-irradiated mice, with a predicted involvement in pathways related to DNA damage repair, hematopoietic, and immune system regulation, suggesting a direct involvement of these pathways in mediating radiation-induced systemic effects. In conclusion, we proved the role of EVs in transmitting certain radiation effects, identified miRNAs carried by EVs potentially responsible for these effects, and showed that the pattern of changes was often different in the directly irradiated and EV-recipient bystander mice, suggesting different mechanisms.

## Introduction

The most intensively studied radiobiological consequence of ionizing radiation was for long the induction of DNA damage and cell death as well as the various cellular pathways activated in response to DNA damage in the directly irradiated cells. The discovery of non-targeted effects of irradiation, including genomic instability and bystander effects, have shifted the focus of radiobiological research from a purely DNA target-based orientation to a much more dynamic science where cellular responses, micro/macro-environmental influences, and systemic effects are at least as important as the dose directly absorbed by the cells and the organism (1, 2). Radiation-induced activation of pro- or anti-inflammatory pathways is a radiation response mechanism equally important at systemic level as DNA damage response at cellular level. Therefore, molecular pathways connecting radiation with inflammatory and immune responses are intensively studied. In a recent meta-analysis, several genes and pathways involved in immune response following ionizing radiation (IR) exposure were identified, such as transforming growth factor beta (TGFβ) signaling pathway, interleukin pathways, nuclear factor kappa B (NFκB) as the key transcription factor in the activation of immune system by IR, as well as regulation of DNA damage response by microRNAs (miRNA) (3). The multiple ways of the initiation of an immune response by radiation exposure was recently reviewed by Candeias and Testard. The authors highlight the importance of toll-like receptors (TLRs) and the direct activation of inflammatory cytokine genes by NFκB and p53 (4).

Radiation-induced bystander effects (RIBE) develop in cells which are not directly hit by IR as a result of signals received from directly irradiated cells. These effects can be classified as local, manifesting within 5 mm from the directly targeted cells and distal when bystander signals are transmitted to distances greater than 5 cm from the directly irradiated cells. These latter effects can be considered as systemic bystander effects (5). RIBE consist of DNA damage, alterations in gene expression, apoptosis, cell death, or genomic instability (6–10). It has been shown that RIBE manifest even at low doses of radiation (11) and that bystander signals can be transmitted both *via* gap junctions and soluble factors, such as TGFβ, IL6, IL8, tumor necrosis factor alpha (TNFα), reactive oxygen species (ROS), or miRNA released into the extracellular environment (12–14). A detailed overview of existing literature data about mediators of local and systemic bystander effects as well as mechanisms how RIBE develop has been recently published (5). The *in vivo* studies related to immune responses elicited by direct radiation and bystander signals have been recently rewieved by Hekim et al. also, listing many important pathways mediating T-cell activation (or suppression), antigen-presenting cell, and natural killer (NK) cell activation (15).

Extracellular vesicles (EVs) are membrane-coated bodies actively released by various cell types. Based on their size distribution and biogenesis, EVs are divided into exosomes (released by multivesicular bodies upon cellular membrane fusion with a diameter of 50–100 nm), microvesicles (MVs) (formed by membrane budding with a diameter of 20–1,000 nm), and apoptotic bodies (released during apoptosis with a diameter of up to 5,000 nm) (16, 17). EVs have important roles in intercellular communication by transferring genetic material (in the form of mRNA and miRNA) and various proteins both to neighboring and distant recipient cells (18), thus influencing their function. Mounting evidences suggest that EVs may be involved in RIBE (19–22) albeit all of these evidences are restricted to *in vitro* studies.

The bone marrow (BM) is a particularly radiosensitive organ where apart from the hematopoietic stem cells and progenitor cells, there is also the stroma composed of fibroblasts, endothelial cells, mesenchymal stem cells, osteoblasts, osteoclasts, adipocytes, and chondrocytes. A close and dynamic cooperation exists between the hematopoietic stem cell compartment and BM stroma, which maintain and adapt to the needs of hematopoiesis and tissue turnover (23). At higher doses where direct effects dominate, the damage of the stem cells determines both the level of BM damage and the long-term health consequences. At lower doses, where radiation-induced direct cell death is moderate and bystander effects are prevalent, bystander signaling between the two compartments might significantly influence BM damage, with an impact on long-term health outcomes.

**Figure 1:**
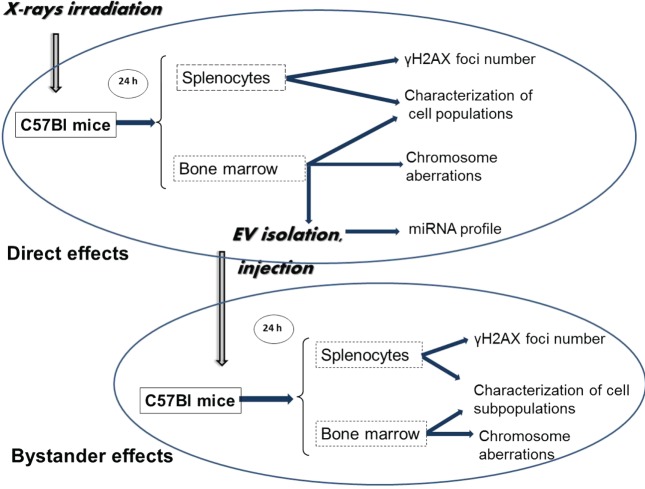
**Schematic representation of the workflow of the study**. C57Bl/6 mice were irradiated with different doses of ionizing radiation (0–2 Gy). Mice were sacrificed 24 h later; spleen and bone marrow (BM) were collected. Extracellular vesicles (EVs) were isolated from the BM supernatant. Bystander effects were monitored by injecting the BM-derived EVs in non-irradiated healthy mice, and 24 h later, the same organs were harvested as from the directly irradiated animals. DNA double-strand break analysis was performed by γ-H2AX assay from the spleen cells, chromosomal aberration were evaluated from the BM cells. BM and spleen cells were characterized phenotypically. EVs from BM of directly irradiated animals were subjected to miRNA profiling.

In the present study, we have investigated the role of BM-derived EVs in mediating systemic RIBE *in vivo*. EVs isolated from the BM of irradiated mice were transferred intravenously into healthy naïve animals. The effects of EV transfer were followed on the BM cells and splenocytes of EV-recipient mice (called bystander mice) (Figure 1). We have found that transfer of EVs from irradiated mice induced various effects in the recipients. Alterations in the recipient mice resembled the alterations exhibited in the directly irradiated animals, suggesting that EVs could transmit biological information from irradiated to unirradiated cells. We also analyzed the miRNA cargo of the EVs prepared from the BM of directly irradiated mice and identified a panel of differentially expressed miRNA suggesting their involvement in mediating RIBE.

## Materials and Methods

### Animal Model and Irradiation

Nine- to fourteen-week-old male C57/BL6 mice were used in all experiments. Mice were kept and investigated in accordance with the guidelines and all applicable sections of the Hungarian and European regulations and directives. This study was carried out in accordance with the recommendations of the 1998 XXVIII Hungarian law about animal protection and welfare. All animal studies were approved, and permission was issued by Budapest and Pest County Administration Office Food Chain Safety and Animal Health Board. Mice were total-body irradiated with 0 (control), 0.1, 0.25 and 2 Gy X-rays using THX-250 therapeutic X-ray source (Medicor, Budapest, Hungary). For each dose, 12–15 mice were used. Mice were selected from at least five different litters, which were mixed prior to irradiation or bystander injections, so that each experimental group randomly contained mice aged between 9 and 14 weeks.

### Isolation of Murine BM Cells and Splenocytes

Bone marrows were isolated from the femur and tibia of mice by flushing out the tissue from the diaphysis of the bones and suspended in phosphate-buffered saline (PBS). BM single-cell suspension was made by mechanical disaggregation of the tissue. Intact, viable cells were pelleted by centrifugation at 500 *g*, 4°C for 10 min. Part of the pelleted BM cells was processed freshly for phenotypical characterization by flow cytometry while another part was suspended in heat-inactivated fetal bovine serum containing 10% dimethylsulphoxide, frozen in liquid nitrogen, and sent to Oxford Brookes University for chromosomal analysis. The BM supernatant was used for EV isolation.

Spleens were mechanically disaggregated and cell suspensions were collected and pelleted in PBS. Red blood cells were removed by incubation of the pellets in 5 ml lysis buffer containing 1.66% ammonium chloride for 5 min. Cells were washed with PBS and passed through a 40-µm cell strainer to obtain single-cell suspension.

Live BM and spleen cells were counted by trypan blue exclusion. Cells were used for subsequent immune phenotyping of different subpopulations, apoptosis, and γ-H2AX staining.

Bone marrow cells and spleens of irradiated and bystander mice were processed individually.

### Isolation, Validation, and *In Vivo* Transfer of EVs

Extracellular vesicles were prepared from BM supernatant of control and irradiated animals by pooling the BM supernatant from a minimum of eight mice/radiation dose. EVs were isolated 24 h after irradiation by the ExoQuick-TC kit (System Biosciences, Palo Alto CA, USA), following the manufacturer’s instructions. Briefly, the supernatant was pooled and incubated overnight at 4°C with ExoQuick-TC solution followed by centrifugation at 1,500 *g* for 30 min. EV pellets were suspended in 200 µl PBS. A GE Healthcare PD SpinTrap G-25 desalting column (GE Healthcare, Life Sciences, WI, USA) was used to remove ExoQuick polymers from the EV solution.

The hydrodynamic size of EVs was determined by the dynamic light scattering (DLS) method using an Avid Nano W130i DLS instrument (Avid Nano, High Wycombe, UK).

For transmission electron microscopy, EV samples kept in 3% PFA were applied to copper grids and negatively stained with a 0.5% uranyl acetate (v/v) solution for 2 min. Grids were air dried for 10 min and viewed using a Hitachi H-7650 transmission electron microscope (Hitachi Ltd., Tokyo, Japan) operated at 100 kV.

Protein content of EVs was measured by Bradford protein assay kit (Thermo Fisher Scientific, Waltham, MA, USA) using a Synergy HT (Biotek, Winooski, USA) plate reader.

For Western blot analysis of exosome-specific protein markers, EVs were lysed with RIPA lysis buffer containing 2% protease inhibitors (Sigma-Aldrich, Darmstadt, Germany). Equal amounts of protein lysates from the EVs prepared from BM of mice irradiated with different doses were loaded and electrophoresed on 10% sodium dodecyl sulfate-polyacrylamide (SDS-PAGE) gel and transferred to PVDF membranes (Bio-Rad, Hercules, CA, USA). Murine BM whole cell lysate treated in the same way was used as control. As a protein standard, Prism Ultra Protein Ladder (Abcam) was used. Anti-mouse CD9, TSG101, and calnexin antibodies (Abcam) were diluted as suggested by the supplier, and lysates were incubated at room temperature (RT) for 1.5 h, followed by 1-h incubation with horseradish peroxidase-conjugated goat anti- rabbit secondary antibody (Abcam). Membranes were washed in Tris-buffered saline-tween buffer three times, and protein bands were visualized using 3,3′-diaminobenzidine substrate (Sigma-Aldrich), by chromogenic method.

Extracellular vesicle-associated acetylcholinesterase activity was determined in EV solution using the Acetylcholinesterase (AchE) Assay Kit (Abcam, Cambridge, UK) over a time period of 30 min by following absorbance at 410 nm with a Synergy HT plate reader.

For setting up the bystander animals, EVs isolated from the directly irradiated animals were injected in the tail vein of healthy unirradiated mice, using 10 µg of EVs suspended in 100 µl PBS. Mice were sacrificed 24 h after EV injection. BM and spleen from the bystander animals were isolated as described above for the directly irradiated animals and used for immune phenotyping and DNA damage assay.

### Immunostaining of Murine Splenocytes for γ-H2AX Assay

γ-H2AX assay was performed from the freshly isolated splenocytes of the directly irradiated and bystander animals both by immunocytochemistry and flow cytometry. For each sample 10^6^ cells in 500 µl PBS were seeded on 13-mm round coverslips placed in 24 well plates. Plates were centrifuged at 35 *g* (500 rpm) for 5 min, supernatant was removed, and cells were fixed in 0.5 ml 2% paraformaldehyde (PFA) at RT for 5 min. Wells were washed with PBS under low-speed shaking three times 5 min. Permeabilization was performed at RT for 15 min using 0.5 ml 0.25% Triton-X 100 solution with 0.1% glycine. After subsequent washing and 3% bovine serum albumin (BSA) blocking (30 min, RT), incubation with primary antibody against γ-H2AX [phospho-Histone-H2A.X (Ser139) (20E3) rabbit monoclonal antibody (mAb), Cell Signaling Technology, Leiden, The Netherlands] was performed at RT for 40 min. This was followed by staining with Alexa 588-conjugated goat anti-mouse secondary antibody (Abcam) at RT for 30 min. After three consecutive washing steps, coverslips were removed from the plate and mounted onto a microscope slide using one drop of Fluoroshield mounting medium with DAPI (Abcam). For quantitative analysis, foci were manually counted using a Zeiss Axio Imager A1 phase-contrast fluorescent microscope (Carl Zeiss microscopy, GmbH, Oberkochen, Germany) equipped with a 100× objective. Images were analyzed by the Zen2012 software (Carl Zeiss microscopy, GmbH). At least 100 randomly chosen cells or 50 foci per slide were counted.

For the analysis of γ-H2AX by flow cytometry, splenocytes were fixed in 4% PFA at 37°C for 10 min. Permeabilization was done in 90% ice-cold methanol for 30 min. Labeling with primary and secondary antibodies was performed as above. The proportion of γ-H2AX-positive cells was determined using a FACSCalibur flow cytometer (Beckton Dickinson, NJ, USA). Analysis was performed using the Cell-Quest Pro data acquisition and analysis software (Beckton Dickinson).

### Quantification of Chromosomal Aberrations

Frozen BM cell pellets were thawed, washed two times with MEM-α medium, and cells were pelleted again for chromosome analysis by centrifugation at 180 *g* for 8 min at RT. Supernatants were removed, and cell pellets were resuspended prior to addition of fresh MEM-α media (Thermo Fisher Scientific) and 10 µg/ml demecolcine (Sigma-Aldrich). Tubes were then placed for 1 h in a humidified 5% CO_2_ incubator at 37°C followed by centrifugation for 10 min at 200 *g* RT. Supernatants were discarded, and the cell pellets were each resuspended in 5 ml of 74 mM potassium chloride solution (VWR International, Radnor, USA) and incubated for 30 min at 37°C in a water bath. To each tube, 3 ml of “½ strength hypotonic solution” [1.94 mM Tri-sodium citrate solution (VWR) and 3.75 mM potassium chloride solution (VWR)] was added, and further incubated for 8 min. Cells were fixed in 3:1 Carnoys fixative (Fisher Scientific, Hampton, New Hampshire, USA) for 13 min. Samples were centrifuged at 200 *g* for 10 min at RT, pellets resuspended again in fixative and incubated for 30 min at RT prior to centrifugation at 200 *g* for 10 min at RT, and the procedure was repeated once more with 20 min incubation. Cells were kept at −20°C overnight.

Slides were prepared from the fixed samples as follows: samples were centrifuged at 180 *g* for 10 min, supernatants were aspirated, and pellets resuspended in approximately 2 ml of fresh 3:1 fixative. Single-use fine-tip minipastettes (Alpha Laboratories Ltd., Eastleigh, Hampshire, UK) were used to pipette each cell suspension up and down before dropping a single drop onto the center of individual labeled degreased microscope slides. This process of layering cells was repeated until there was a reasonable coverage of cells on each microscope slide. Depending on the sample’s mitotic index, two to four slides were prepared from each sample. Samples were then air dried at RT for 24 h prior to staining with 6.7% Giemsa Stain improved R66 solution Gurr^®^ (VWR) in buffer solution (pH 6.8). Slides were air dried before addition of cover slips secured with Entellan^®^ new rapid mounting media (VWR) and coded for analysis. Where possible, 200 well spread metaphases were analyzed from each sample using a light microscope and 100× objective.

The Fisher’s exact test was performed, each irradiated/bystander group were compared to their respective control. Groups with *p*-values less than 0.05 were considered statistically significant.

### Immune Phenotyping of Splenocytes and BM Cells

The following directly labeled anti-mouse monoclonal antibodies were used for BM cell phenotypical analysis: CD90.2-APC and CD45-PE/Cy7 for lymphoid progenitors, CD61-APC and CD41-FITC for megakaryocytic population, CD71-PE and Ter119-FITC for erythroid precursors, CD11b-PE and Gr1-FITC for granulocytes/monocytes progenitors, Lineage Cocktail (CD3, Gr1, CD11b, CD45R, Ter119)-FITC, Sca1-PE, cKit (CD117)-APC for hematopoietic stem cells, all purchased from BioLegend (BioLegend, San Diego, CA, USA).

The phenotypical analysis of splenocytes was performed using the following anti-mouse antibodies: CD4-PE/Cy5, CD8a-PE (BioLegend) for helper and cytotoxic T cells, CD19 (BioLegend) for B cells, CD11c-PE, I-Ab-FITC, and TLR4 (CD284)-PE/Cy7 (all from BioLegend) for dendritic cells (DCs), and NK1.1-FITC (BioLegend) for NK cells. To detect proliferative cells, Ki67-eFluor660 (eBioscience, San Diego. USA) was used.

Single-cell suspensions of splenocytes or BM cells were incubated with the fluorescently labeled antibodies in PBS containing 1% BSA, at 4°C for 20 min for cell surface staining. For intracellular staining (Ki67), cells were permeabilized using the Foxp3 Fix/Perm Buffer (eBioscience), according to the manufacturer’s instructions. Measurements were performed with a FACSCalibur flow cytometer as described above.

### Analysis of Apoptosis in Irradiated and Bystander Splenocytes

Apoptosis was detected by the TUNEL assay using the Mebstain Apoptosis Kit Direct (MBL, Nagoya, Japan). Briefly, splenocytes were kept in 250 µl ice-cold PBS and 750 µl 75% ethanol at 4°C for 20 min. Cells were washed, pelleted, and resuspended in the residual PBS. Fixation was done with 1 ml 1% PFA at RT for 30 min. Fixed cells were kept at 4°C overnight and then pelleted, and a mix of 27 µl of terminal deoxy-nucleotidil transferase (TdT) buffer/1.5 μl of FITC-dUTP/1.5 μl TdT enzyme per sample was added to the pellet. FACS analysis was performed after incubating the samples at 37°C for 60 min.

### Profiling of miRNA Isolated from BM-Derived EVs

#### miRNA profiling

Extracellular vesicles were prepared from BM of control and irradiated mice by pooling the BM supernatant of five mice/radiation dose/experiment. Three independent experiments were performed.

The EVs prepared were sent for analysis to Exiqon Services (Exiqon Services, Vedbaek, Denmark), where RNA isolation, miRNA profiling with a polymerase chain reaction (PCR) panel, and data pre-processing were performed.

Total RNA was extracted by Exiqon from the EVs using the Qiagen miRNeasy^®^ Mini Kit (Qiagen, Hilden, Germany). Briefly, EVs were lysed in Qiazol lysis reagent then the lysate was incubated with chloroform at RT for 2 min. The supernatant was treated with 100% ethanol and centrifuged using a Qiagen RNeasy^®^ Mini spin. The Qiagen RNeasy^®^ Mini spin column was rinsed with the provided buffers then transferred to a new microcentrifuge tube, and the lid was left uncapped for 1 min to allow the column to dry. Total RNA was eluted with 50 µl of RNase-free water.

MicroRNA analysis with RT-PCR array was also performed by Exiqon. Briefly, 19 µl RNA was reverse transcribed in 95 µl reaction volume using the miRCURY LNA™ Universal RT microRNA PCR, polyadenylation, and cDNA synthesis kit (Exiqon). cDNA was diluted 50× and assayed in 10-µl PCR reaction volume according to the protocol of the kit; each miRNA was assayed once by qPCR on the miRNA Ready-to-Use PCR, Mouse&Rat panel I + II using ExiLENT SYBR^®^ Green master mix. Negative controls excluding template from the reverse transcription reaction were performed and profiled similarly to the samples. The amplification was performed in a LightCycler^®^ 480 Real-Time PCR System (Roche, Basel, Switzerland) in 384 well plates. The amplification curves were analyzed using the Roche LC software, both for determination of quantification cycles (Cq) (by the second derivative method) and for melting curve (T_m_) analysis.

The amplification efficiency was calculated by Exiqon using algorithms similar to the LinReg software. All assays were inspected for distinct melting curves, and the T_m_ was checked to be within known specifications for the assay. Furthermore, assays must have been detected with three Cqs less than the negative control, and with Cq < 37 to be included in the data analysis. Data that did not pass these criteria were omitted from any further analysis. Cq was calculated as the second derivative.

Using NormFinder, the best normalizer was found to be the average of assays detected in all samples. All data were normalized to the average of assays detected in all samples (average − assay Cq). The heat map diagram and the principal component analysis (PCA) were performed on all samples and on the top 50 miRNA with highest SD. The normalized Cq values have been used for the analysis.

#### Data Analysis of miRNA Arrays

Data analysis of the miRNA arrays, based on normalized Cq values (determined by Exiqon) was performed by our group. For defining differentially expressed miRNA, differences were calculated pairwise as fold changes compared to the miRNA expression from non-irradiated (0 Gy) samples. The average fold changes of the three independent experiments were calculated. Student’s paired *t*-test was applied to these data for significance analysis.

To uncover the potential biological function of miRNAs differentially expressed in EVs both in 0.1 Gy and 2 Gy irradiated animals, a multiple miRNA effect analysis using DIANA-miRPath v.3.0 software (24) was performed. The DIANA-microT-CDS target prediction algorithm was employed to predict miRNA targets. This was combined with Kyoto Encyclopedia of Genes and Genomes (KEGG) and Gene Ontology (GO) databases. A target prediction threshold of 0.8 with *p*-value of 0.05 and false discovery rate correction was applied. A list of the predicted target genes of miRNAs altered by IR and a list of KEGG pathways ranked by significance was obtained in this way. Next, seven pathways closely connected to our investigated functional endpoints and considered by us as the most important ones were chosen from the KEGG pathway list and mapped those genes from the list of predicted target genes into the selected KEGG pathways, which were targeted by more than one differentially expressed miRNAs.

To identify the processes co-regulated by these genes, a global network of functional coupling was constructed using FunCoup 3.0 software with the focus on finding new couplings between search terms. Within this software, an expansion algorithm was used with genes as a group, prioritizing common neighbors (meaning that all links to all the genes of interest are considered and genes that are most strongly linked to other genes of interest are prioritized). A confidence threshold of 0.8 and expansion depth of one step including 20 nodes per expansion step was applied during analysis.

### Statistical Analysis

Data are presented as mean ± SD. In most of the cases Student’s *t*-test was applied to determine statistical significance, using GraphPad Prism version 6.00 for Windows (GraphPad Software,^1^ La Jolla, CA, USA), if not stated otherwise. Data were considered statistically significant if *p*-value was lower than 0.05.

## Results

### BM-Derived EVs Are Composed of Exosomes and MVs

**Figure 2:**
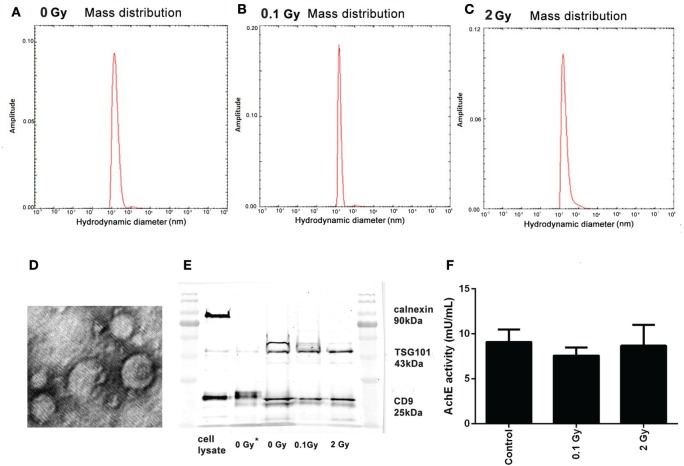
**Characterization of bone marrow-derived extracellular vesicles (EVs)**. **(A–C)** Size distribution of the EVs isolated 24 h following irradiation with 0 Gy **(A)**, 0.1 Gy **(B)**, and 2 Gy **(C)**, determined by measuring the hydrodynamic size using the dynamic light scattering method. **(D)** Transmission electron microscopy imaging of EVs. Representative image of EVs isolated from control (0 Gy) mice. **(E)** Western blot analysis of EVs for calnexin, TSG101, and CD9. Lanes 1 and 7 show the protein ladder, lane 2 is the cell lysate, lane 3 is an unirradiated (0 Gy) sample isolated with Exoquick-TC, lanes 4–6 are 0, 0.1, and 2-Gy samples isolated with Exoquick-TC and filtered through PD SpinTrap G-25 column **(F)** Acetylcholinesterase enzyme activity of EVs from samples irradiated with different doses was assessed by an enzyme activity assay. OD was measured at 412 nm. Data are the mean ± SD of three independent experiments.

The measured hydrodynamic mean diameter of the EVs was 169 nm (±SD = 83), 252 nm (±SD = 136), and 226 nm (±SD = 106) in mice treated with sham, 0.1 Gy, and 2 Gy irradiation, respectively. Differences in the mean diameter of EVs were statistically not significant, indicating that irradiation did not alter the size distribution of the EVs (Figures 2A–C).

Electron microscopic analysis indicated the presence of vesicular structures in the isolates, many of which had a typical “cup-shaped” aspect characteristic for exosomes (Figure 2D).

The EVs were further characterized by Western blot analysis following minimal required criteria suggested by Lötvall et al. for EV identification (25): a minimum of two EV-specific protein markers expected to be present in EV isolates and an endosomal protein not expected to be present in EVs were determined. EVs from both control and irradiated mice were positive for two markers commonly used for exosome identification: the tetraspanin CD9, a protein highly enriched in EVs (26), and the TSG101, involved in multivesicular biogenesis (27), and were negative for calnexin, an endoplasmic reticulum marker (Figure 2E). The coexistence of these criteria is considered as EV markers and identifies our isolated samples as EVs.

The AchE activity was also measured in the isolated EVs. Although AchE activity is not considered as an absolute specific EV marker, if present, it can further strengthen their identity. AchE activity was present in comparable amounts in the EV isolates from control and irradiated animals confirming the presence of exosomes and MVs in all samples (Figure 2F).

### *In Vivo* Transfer of EVs from Irradiated Mice Induces γ-H2AX Foci Formation in the Spleen of Recipient Mice

**Figure 3:**
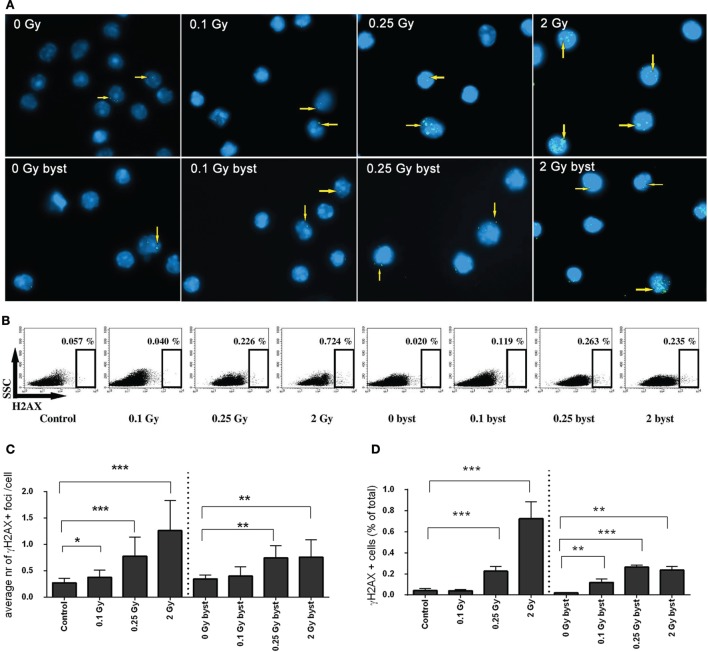
**DNA double-strand breaks in directly irradiated and bystander animals measured by γ-H2AX assay**. **(A)** Microscopic image of splenocytes of directly irradiated and bystander animals immunostained for γ-H2AX. Arrows indicate the location of γ-H2AX^+^ foci, cell nuclei stained with DAPI are shown in blue, γ-H2AX^+^ foci stained with Alexa488 are shown in green. **(B)** Flow cytometry plots of splenocytes stained for γ-H2AX. The gates indicate the percent of γ-H2AX^+^ cells within the splenocytes. **(C)** Histogram representing the average number of γ-H2AX^+^ foci per cells counted by fluorescent microscopy (*N* = 7–10). **(D)** Histogram representing the percent of γ-H2AX^+^ cells within the splenocytes measured by flow cytometry (*N* = 7–10). Bars represent mean ± SD, significance was tested by Student’s *t*-test (*p < 0.05, **p < 0.01, ***p < 0.001).

The frequency of DNA double-strand breaks (DSBs) generated by *in vivo* transfer of EVs was investigated in the spleen of bystander animals and compared to DSBs generated in total-body irradiated mice. DSB analysis was performed by the γ-H2AX assay using both a fluorescent microscopy and a flow cytometry protocol. The fluorescent microscopy protocol is considered a more accurate and more specific method than evaluating the frequency of event-positive cells by flow cytometry (28). However, the latter method is much quicker, allows the quantification of much higher number of cells, and in this way, increases the statistical power in cases where the number of alterations is low (29). As expected, a dose-dependent increase of DNA damage was detected in directly irradiated animals. In bystander mice, which received EVs from irradiated animals γ-H2AX foci levels also increased both in terms of average foci/cell (Figures 3A,C) and the frequency of γ-H2AX-positive cells (Figures 3B,D). However, the increase was more moderate than in the directly irradiated animals, and no strict dose-dependency was observed, since the detected damage levels after low- and moderate-dose irradiation were comparable to high-dose irradiation (Figure 3). These data indicate that BM-derived EVs originating from irradiated animals could mediate the activation of the DNA damage response pathway in the splenocytes of EV-injected bystander animals and that RIBE peaked at low doses.

### EV Transfer from Irradiated Mice Induces Chromosomal Aberrations in Recipient Animals

**Figure 4:**
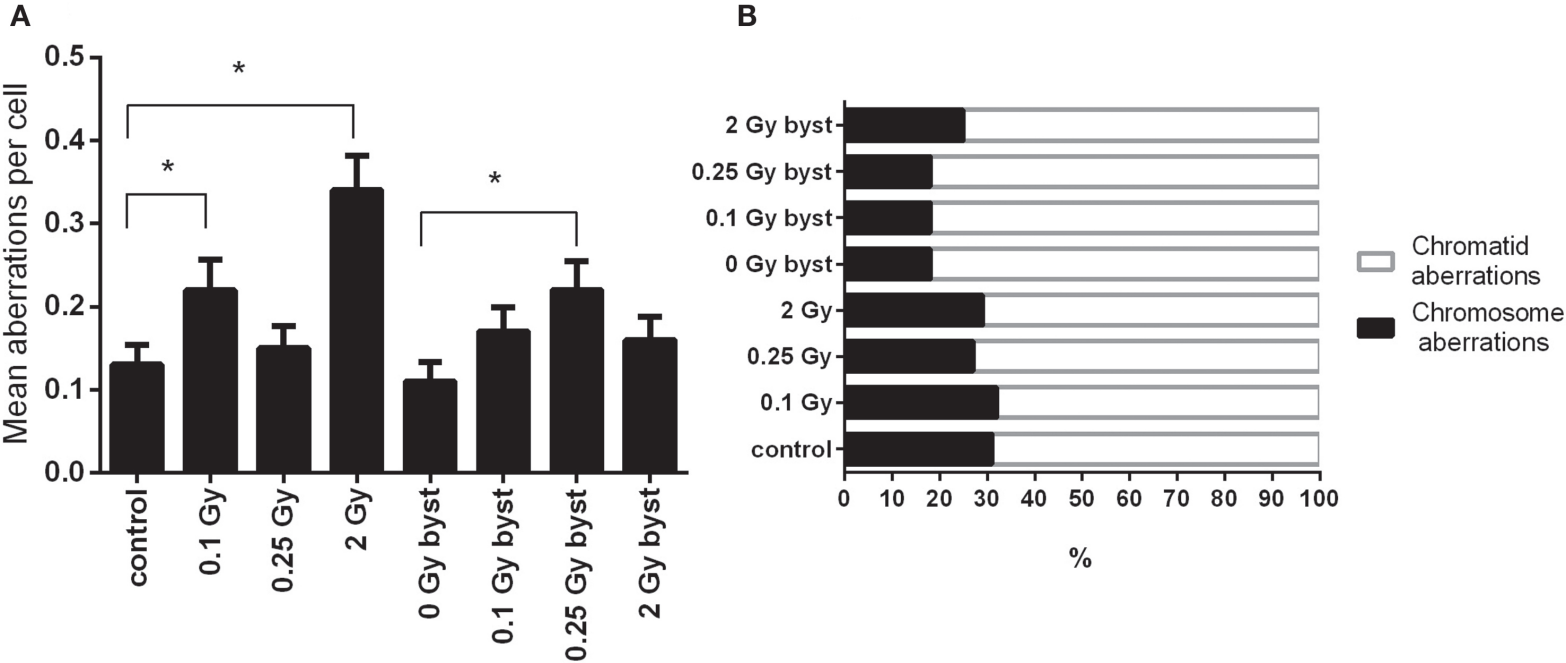
**Chromosomal aberrations in bone marrow cells isolated from irradiated and control mice**. **(A)** Chromosomal aberrations were scored in 200 metaphase spreads 24 h after irradiation or extracellular vesicles transfer. Bars represent mean ± SEM, significance was tested by Fisher’s exact test. Each irradiated/bystander group was compared to its respective control. Groups with *p*-values less than 0.05 were considered statistically significant (*). **(B)** Total aberrations were scored regardless of their nature and plotted as fractions of the total.

As expected, the frequency of chromosomal aberrations increased in the BM cells of directly irradiated mice. In bystander mice which received EVs from directly irradiated animals, the frequency of chromosomal aberrations also increased, but to a lesser extent. In the directly irradiated mice, the highest level of chromosomal aberrations was detected at the highest dose, while in the bystander mice it peaked around 0.25 Gy (Figure 4A). Most aberrations detected were chromatid in nature (Figure 4B). EV-recipient bystander groups overall showed a greater proportion of chromatid aberrations compared to directly irradiated mice.

### EV Transfer from Irradiated to Bystander Mice Induces Quantitative Changes in the Cellular Composition of BM and Spleen

#### Alterations in BM

Direct as well as EV transfer-induced bystander effects were studied in more detail in the BM stem and progenitor cell compartments. Namely, alterations in the hematopoietic stem cells (Lineage-Sca-1^+^cKit^+^), lymphoid progenitors (CD45^+^CD90.2^+^), myeloid progenitors (Gr1^+^CD11b^+^), megakaryocytes, and megakaryocyte progenitors (CD41^+^CD61^+^), as well as erythroid progenitors (CD71^+^Ter119^+^) were studied.

**Figure 5:**
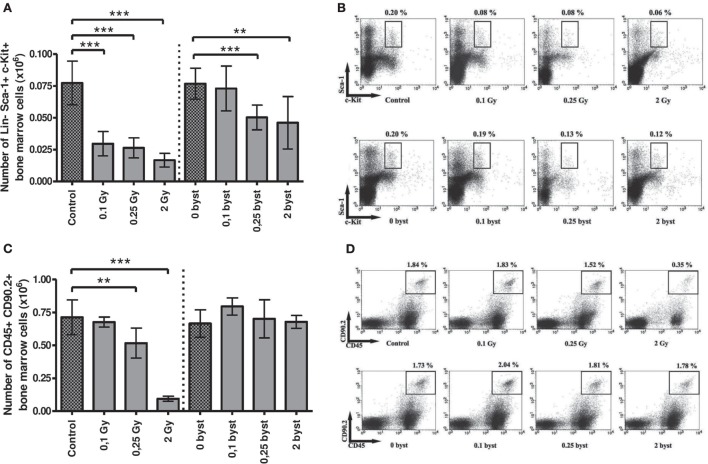
**Immune phenotyping of bone marrow (BM) cells isolated from irradiated and bystander animals**. BM cells isolated from directly irradiated and bystander mice were stained with fluorescently labeled antibodies and were analyzed by flow cytometry. Histogram **(A)** represents the cell number of hematopoietic stem cells in mouse BM. Dot plots from the flow cytometric analysis **(B)** represent the distribution of hematopoietic Sca-1 and c-Kit (CD117) double positive, Lineage (CD3^+^, Gr1^+^, CD11b^+^, CD45R^+^, Ter119^+^) negative stem cells. The plots show the gated Lineage negative cells in which Sca1^+^c-Kit^+^ cells were evaluated. Histogram **(C)** shows the number of lymphoid progenitors in mouse BM. Dot plots from the flow cytometric analysis **(D)** show the distribution of CD45 and CD90.2 double positive lymphoid progenitor cells. Bars represent mean ± SD (*N* = 7–12), significance was tested by Student’s *t*-test (*p < 0.05, **p < 0.01, ***p < 0.001).

In directly irradiated mice, the absolute number of the hematopoietic stem cells decreased to 38, 34, and 21 after 0.1, 0.25, and 2 Gy irradiation, respectively, when compared to unirradiated animals (Figures 5A,B). In the EV-recipient animals the stem cell numbers also decreased, but changes were milder and moderately depended on dose. In the 0.1-Gy bystander group, changes were statistically not significant, while in the 0.25- and 2-Gy bystander mice, the number of hematopoietic stem cells decreased to almost identical levels (65 and 60% surviving cells) (Figures 5A,B).

Beside stem cells, the lymphoid progenitors were another radiosensitive population, since their number decreased to 70 and 15% after 0.25 and 2 Gy, respectively, in directly irradiated mice. However, in this case, EV could not transmit the effect to recipient mice (Figures 5C,D). Megakaryocyte progenitors in BM of directly irradiated animals exhibited a small decrease in cell number. Although changes were statistically not significant, the tendency showed a dose-dependent decrease (Figure S1A in Supplementary Material). Myeloid and erythroid progenitor cell numbers were not affected either by irradiation or EV transfer (Figures S1B,C in Supplementary Material).

#### Alterations in the Spleen

Lymphocytes constitute the major cellular fraction within the murine spleen (approximately 85%), which is also a rich source of DCs. Radiation-induced direct and bystander changes were monitored by following the absolute number and proliferative capacity of the different lymphocyte subpopulations (CD4^+^ and CD8^+^ T lymphocytes, CD19^+^ B cells, and NK cells), as well as the number and activation status of splenic DCs.

**Figure 6:**
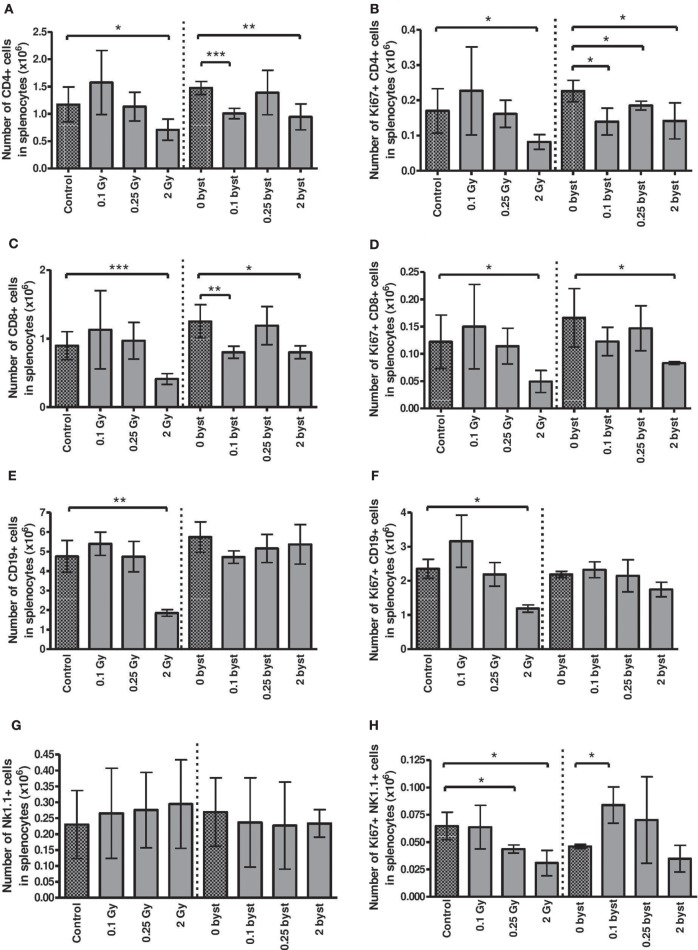
**Immune phenotyping of splenic lymphocytes isolated from irradiated and bystander animals**. Splenocytes isolated from directly irradiated and bystander mice were stained with fluorescently labeled antibodies and were analyzed by flow cytometry. Histograms represent the cell numbers of the corresponding subpopulations calculated per 10 mg spleen: CD4^+^ T cells **(A)** and proliferating CD4^+^ T cells **(B)**; CD8^+^ T cells **(C)** and proliferating CD8^+^ T cells **(D)**; B cells **(E)** and proliferating B cells **(F)**; natural killer (NK) cells **(G)** and proliferating NK cells **(H)**. Bars represent mean ± SD (*N* = 5); significance was tested by Student’s *t*-test (*p < 0.05, **p < 0.01, ***p < 0.001).

Regarding the direct effect of irradiation on the splenocyte subpopulations, a strong difference in the radiosensitivity of the various cellular subsets was observed. Low doses had no significant effect, but irradiation with 2 Gy reduced CD4^+^ T cell, CD8^+^ T cell, and B cell pool to 60, 45, and 39% of control values, respectively (Figures 6A,C,E). NK cell numbers were not affected by irradiation (Figure 6G). Interestingly, strong bystander responses were detected in CD4^+^ and CD8^+^ T cell populations of animals injected with EVs from irradiated mice, while the effect was absent in B and NK cells (Figures 6A,C,E,G).

The basal proliferation rate was about 9.5, 9, 24 and 26% for CD4^+^, CD8^+^, B, and NK cells, respectively, and EV injection *per se* did not alter this proliferation rate (Figures 6B,D,F,H). Radiation-induced changes in the proliferative capacity of splenocytes reflected their radiosensitivity, decreasing after 2 Gy in all lymphocytes. Bystander responses were similar, albeit milder than in the directly irradiated animals (Figures 6B,D,F,H).

**Figure 7:**
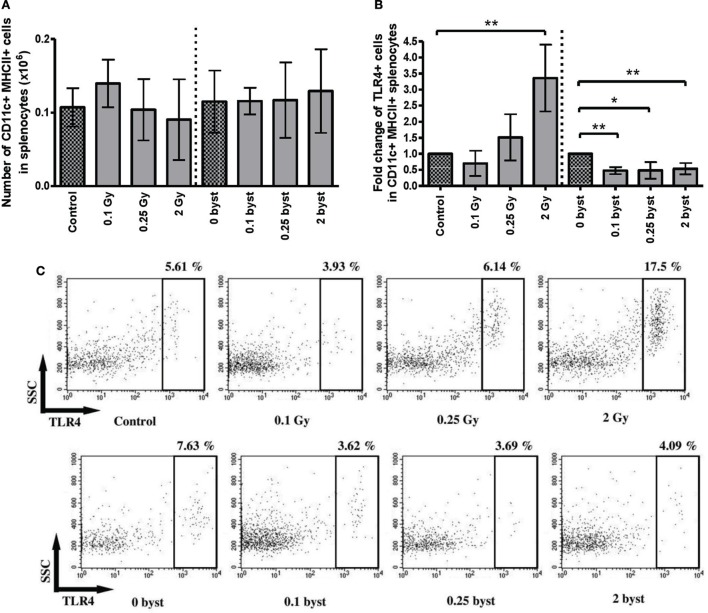
**Immune phenotyping of splenic dendritic cells (DCs) isolated from irradiated and bystander animals**. Splenocytes isolated from directly irradiated and bystander mice were stained with fluorescently labeled antibodies and were analyzed by flow cytometry. Histogram **(A)** shows the number of CD11b^+^ MHCII^+^ splenic DCs per 10 mg spleen. Histogram **(B)** shows the relative ratio of TLR4^+^ dendritic cells within the total splenic DC population. Irradiated and bystander samples were compared to their corresponding unirradiated controls. Dot plots from the flow cytometric analysis **(C)** represent the distribution of TLR4^+^ cells within the DC population. The plots show the gated CD11c^+^ MHCII^+^ positive cells in which TLR4 expression was determined. Bars represent mean ± SD (*N* = 5), significance was tested by Student’s *t*-test (*p < 0.05, **p < 0.01, ***p < 0.001).

Splenic DCs were identified by their CD11c and MHCII double positivity. In contrast to lymphocytes, the number of DCs did not change in the directly irradiated animals. Bystander responses were also absent for all doses (Figure 7A). TLR4 expression on DC cell surface is a sign of DC activation by lipopolysaccharide (LPS) or LPS-like endogenous danger signals, such as high mobility group binding protein 1 (HMGB1) (30). Since radiation-induced cellular damage is associated with danger signal release, we investigated radiation-induced changes in the fraction of TLR4-expressing DCs. A significantly increased fraction of TLR4-expressing DCs was detected after direct irradiation with 2 Gy. Surprisingly, EV-induced bystander responses showed a completely different pattern of TLR4 expression, since the proportion of TLR4-expressing DCs within the total DC population was very strongly reduced after treatment with EVs derived from irradiated animals irrespective of the dose (Figures 7B,C).

### EV Transfer from Irradiated to Bystander Mice Does Not Induce Apoptosis in Splenocytes

Since phenotypical analysis indicated a strong radiation response of splenic lymphocytes both in the directly irradiated and EV-recipient animals, which could be only partially explained by the reduced proliferation capacity of the cells after irradiation, we investigated potential alterations in apoptosis frequency in total splenocytes by the TUNEL assay. As presented in Figure S2 in Supplementary Material, the fraction of apoptotic cells increased strongly in the directly irradiated animals after irradiation with 2 Gy. However, EV-induced bystander responses were completely absent, indicating that EV transfer did not have any apoptosis-inducing effect.

### Analysis of microRNA Profile of EVs Derived from the BM of Irradiated Mice

#### Similar miRNAs Are Affected after Both Low- and High-Dose Irradiation

The average number of miRNAs that could be identified in EVs derived from BM of unirradiated, control mice was 500 per sample. It was not characteristic for irradiation to induce the appearance or disappearance of miRNAs in the EVs with very few exceptions; miRNAs, such as miR-124, miR-346, miR-449c, and miR-381, were present, while miR-695 and miR-761 were absent in the samples irradiated with 2 Gy. Raw data were uploaded to STOREDB database,^2^ accession number [DOI:10.20348/STOREDB/1062], dataset 1101. According to the PCA, samples seemed to cluster based on the radiation group they belonged to, with a better separation of the 2 Gy samples.

**Figure 8:**
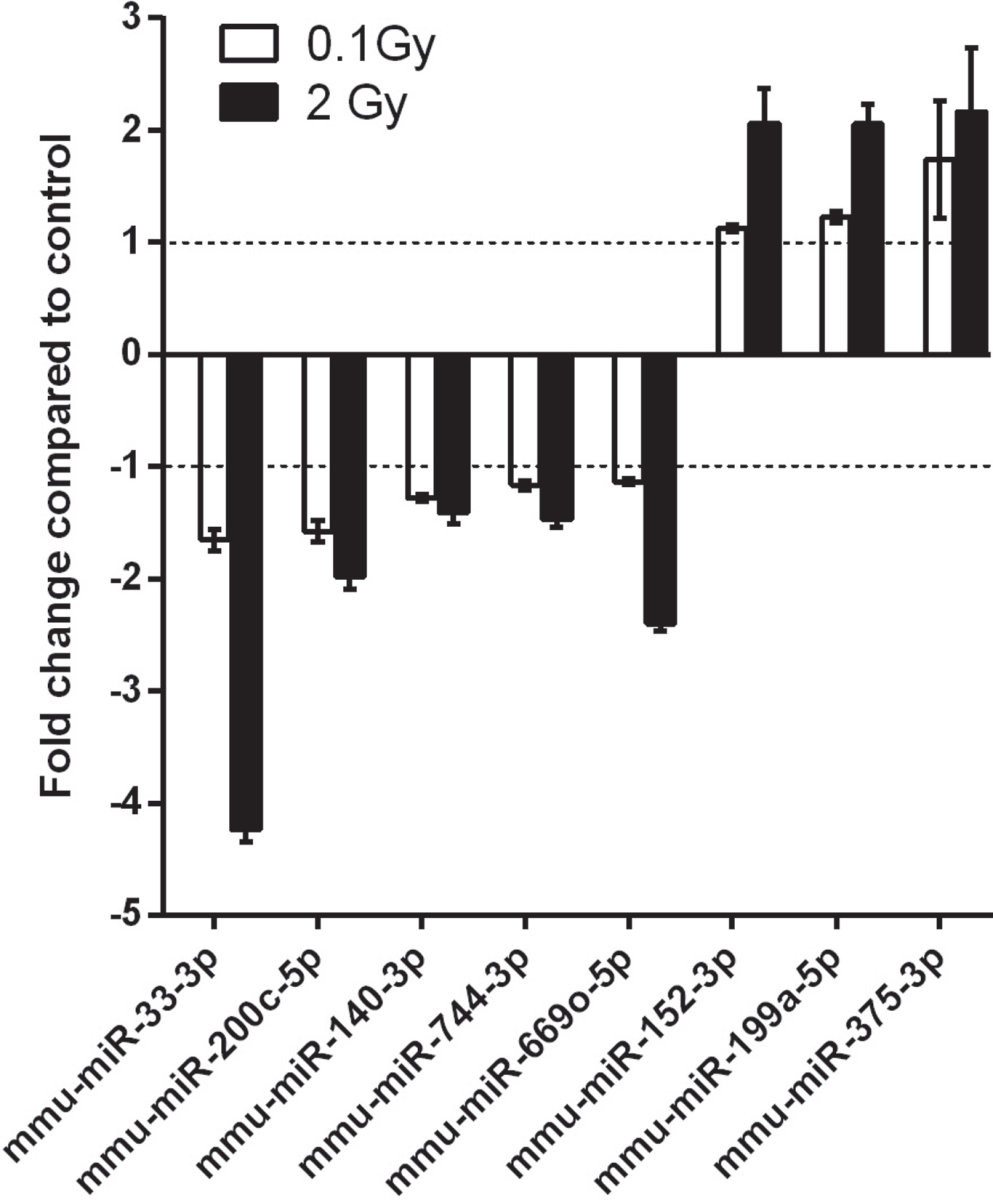
**microRNAs (miRNAs) differentially expressed in both 0.1 and 2 Gy extracellular vesicles (EVs) compared to EVs from control animals**. A miRNA profiling of EVs isolated from bone marrow of control mice and mice irradiated with 0.1 or 2 Gy was performed by a qPCR panel array. miRNAs with significantly modulated expression relative to control are presented in the graph. Data are the mean ± SD of three independent experiments. Significance was tested by Student’s *t*-test (*p < 0.05).

When comparing the miRNA content of the EVs of irradiated and control mice, 20 miRNAs were found to be differentially expressed in the 0.1 Gy group (Table S1 in Supplementary Material) and 90 miRNAs in the 2 Gy group (Table S2 in Supplementary Material) using a *t*-test with a cutoff *p*-value <0.05. Out of these, eight miRNAs were affected after both low- and high-dose irradiation: five miRNAs (mmu-miR-33-3p, mmu-miR-200c-5p, mmu-miR-140-3p, mmu-miR-744-3p, and mmu-miR-669o-5p) were downregulated and three miRNAs (mmu-miR-152-3p, mmu-miR-199a-5p, and mmu-miR-375-3p) were upregulated. Changes in the level of these miRNAs were dose dependent, as shown in Figure 8.

#### miRNA Target Prediction and Pathway Analysis Shows a Direct Link between miRNA Expression Pattern and EV-Induced Changes in the Hematopoietic System after Irradiation

In order to create a link between the differentially expressed miRNAs and EV-induced changes in the hematopoietic system of the bystander animals, a functional analysis using DIANA miRPath software followed by a network analysis using FunCoup 3.0 software was performed.

Analysis of the target genes of the 20 differentially expressed miRNAs in the 0.1-Gy samples revealed that these miRNAs targeted 33 different KEGG pathways (Table S3 in Supplementary Material), whereas the 90 differentially expressed miRNAs derived from the 2-Gy samples targeted 60 KEGG pathways with a high degree of significance (*p* ≤ 0.05) (Table S4 in Supplementary Material).

A more detailed target prediction and pathway analysis of the eight miRNAs modulated in both 0.1 and 2 Gy irradiated samples was performed by applying a GO and KEGG Pathway Enrichment Analysis. While the GO pathway annotates different genes and gene products to certain gross biological terms, such as biological process and subcellular localization (31), the KEGG pathway database is a collection of diagrams representing complex pathway maps of molecular interactions and networks (32). The top GO processes, predicted to be influenced by these eight miRNAs, were associated with development and differentiation, metabolic and biosynthetic processes, cell growth, motility, and cell death. It also showed that all miRNAs within these pathways were located in the following cellular compartments: nuclear chromosome, cytoplasmic stress granule, and cytoplasmic membrane-bound vesicle (Table S5 in Supplementary Material), indicating not only a concentration of IR-induced damage at chromosomal level but also highlighting the vesicular origin of the miRNAs.

**Figure 9:**
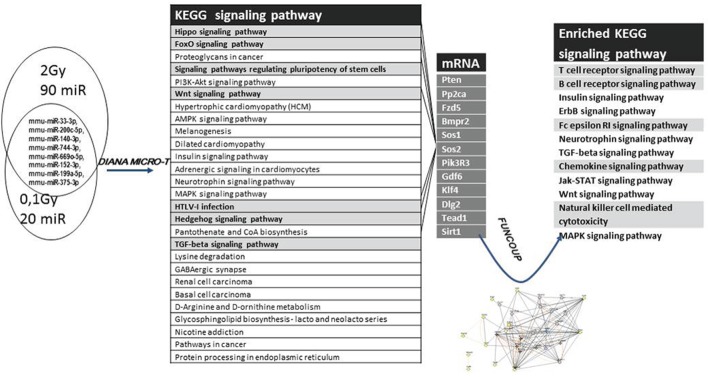
**Analysis strategy for evaluating the effects of differentially expressed microRNAs (miRNAs)**. The set of miRNAs differentially expressed from both 0.1 Gy vs. control and 2 Gy vs. control was analyzed for predicted target genes and predicted pathways using the DIANA miRPath software. Seven pathways (highlighted in gray) considered to be important for the endpoints of the study were further analyzed. Messenger RNAs potentially regulated by more than one differentially expressed miRNA were mapped from these seven pathways, followed by a network analysis of these mRNAs using FunCoup 3.0 software.

Using the KEGG database, 27 pathways were predicted to be influenced by the differentially expressed miRNAs, many of them dealing with mechanisms connected to cellular radiation response, DNA repair [such as Hippo, Hedgehog, Forkhead box O (Foxo), Phosphoinositide-3-kinase (PI3K), TGFβ signaling pathways], as well as pathways connected to the hematopoietic system [signaling pathways regulating pluripotency of stem cells, Wnt signaling pathway, human T cell lymphotrophic virus (HTLV) infections] (Figure 9; Table S6 in Supplementary Material).

**Figure 10:**
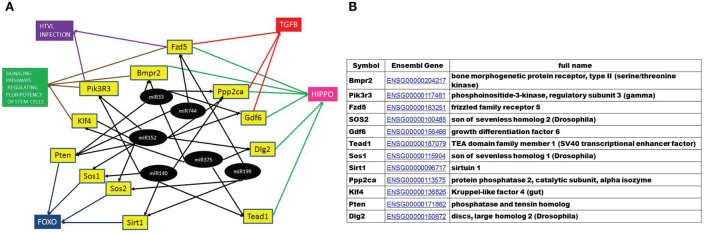
**A hypothetical network of KEGG pathways predicted to be altered by mRNAs targeted by the microRNAs (miRNAs) differentially expressed in the EVs in both 0.1 and 2 Gy samples**. **(A)** Black circles represent differentially expressed miRNA, yellow boxes are their predicted target genes and colored boxes represent the pathways including these genes. **(B)** The full name and annotation of target mRNAs.

To find the putative responsible mRNAs driving the observed functional effects caused by EV transfer (DNA damage and phenotypical changes in central and peripheral hematopoietic system), seven pathways were chosen for further study out of the 27 identified (highlighted in Figure 9). Messenger RNAs potentially regulated by more than one differentially expressed miRNA were mapped from these seven pathways. Twelve mRNAs co-regulated by six miRNAs were found, which were involved in one or more of the selected pathways, as shown in Figures 9 and 10. We also noticed that most of the products of these mRNAs were involved in multiple pathways.

A gene coupling network was constructed by connecting these 12 mRNAs using the FunCoup software. Since FunCoup is a collection of genome-wide functional couplings, which integrates evidence types derived from high-throughput genomics and proteomics data, such as protein–protein interaction, mRNA co-expression, protein co-expression, shared transcription factor binding, and co-miRNA regulation, by shared miRNA targeting (33, 34), it is suitable to reveal new functional links not identified solely by the KEGG pathway analysis. The most enriched signaling pathways detected with this approach were strongly related to the hematopoietic and immune system, such as T cell signaling, B cell signaling, NK-mediated cytotoxicity, chemokine signaling, Fc epsilon signaling, insulin signaling, and Jak–Stat signaling (Figure 9; Table 1).

**Table 1:** **Significantly enriched pathways according to FunCoup network analysis**.

Kyoto Encyclopedia of Genes and Genomes signaling pathway	Number of genes	*p*-Value
T cell receptor signaling pathway	6	5.97E−4
B cell receptor signaling pathway	5	5.97E−4
Insulin signaling pathway	6	5.97E−4
ErbB signaling pathway	5	5.97E−4
Fc epsilon RI signaling pathway	5	5.97E−4
Neurotrophin signaling pathway	5	2.73E−3
TGF-beta signaling pathway	4	6.68E−3
Chemokine signaling pathway	5	1.15E−2
Jak–STAT signaling pathway	4	2.76E−2
Wnt signaling pathway	4	2.76E−2
Natural killer cell-mediated cytotoxicity	4	3.84E−2
MAPK signaling pathway	4	1.02E−1

*Number of genes refers to the number of mRNAs involved in the corresponding pathway*.

## Discussion

Radiation-induced bystander effects have important consequences in radiation protection, since due to this phenomenon not only the directly irradiated cells exhibit biological damage but a significantly larger number of cells are also affected, increasing the likelihood of radiation-induced adverse health effects (35). Therefore, significant effort has been done to understand the mechanisms governing this phenomenon.

Recent works have indicated that EVs released from irradiated cells may play a role in mediating RIBE. Al-Mayah et al. showed that treatment of bystander MCF-7 breast cancer cells with exosomes isolated from media of irradiated cells increased the level of genomic damage (20), and this effect persisted for more than 20 population doublings in the progeny of bystander cells (19). Mutschelknaus et al. demonstrated that exosomes derived from irradiated head and neck cancer cell lines increased both the proliferation and survival of recipient cells (36). We should note, however, that these evidences have been shown exclusively under *in vitro* conditions.

In the present work, we designed an *in vivo* model to study the ability of EVs to mediate bystander effects, where EVs extracted from the BM of total-body irradiated mice were injected intravenously into naïve mice and EV-transmitted effects were followed in the BM and spleen of the EV-recipient animals. The reason for choosing the hematopoietic system for our studies was that both the BM and the spleen are highly radiosensitive tissues, where radiation-induced bystander signals have been identified as important modulators of radiation effects (37, 38). Since it was shown by several research groups that BM was an important tissue milieu where MVs-mediated signals were able to modulate the phenotype of the cells (39–41), we intended to test the hypothesis that EVs could be at least in part responsible for local and/or systemic RIBE. Formerly, we have investigated the *in vivo* biodistribution of BM-derived EVs upon intravenous injection and demonstrated their stable presence both in the spleen and BM 24 h after injection (42). The EVs used for the current experiments had a mean diameter of 200 nm and a cup-shaped aspect and were highly enriched in the TSG101 and tetraspanin CD9 proteins, while lacking cellular markers of endosomal origin, indicating that the EV isolates were composed of exosomes and most probably MVs as well (26, 43). While formerly the exosomes were considered the main and unique EV types involved in intercellular communication, recent publications have proven also the involvement of MVs in this process. MVs, similarly to exosomes, have a rich mRNA, miRNA, and protein cargo (44). Recently, Wen et al. have demonstrated that a combination of exosomes and MVs had a stronger effect in transferring biological processes from one cell to the other than either fraction alone (45).

In order to evaluate the role of EVs in mediating radiation effects, first we investigated whether EVs could transmit systemically radiation-induced DNA and chromosomal damage to unirradiated BM and spleen cells. The most characteristic type of DNA damage caused by IR is DNA DSB, a highly cytotoxic form of DNA damage, which, if not repaired in short time, can lead to cell death or genomic instability (46). The phosphorylation of the histone H2AX in the vicinity of a DSB is considered a specific marker for this type of DNA lesion (47). The phosphorylated H2AX molecules are induced during the repair process of DSB and can be observed as distinct foci in nuclei of the cells in the neighborhood of the damage. The sensitivity of the method to detect even very low doses of radiation exposure was reported in several publications, which also proved that the assay is dose dependent (48–50). In line with these findings, our data showed a correlation of γ-H2AX-positive cells with the applied radiation dose within the spleens of the directly irradiated animals.

γ-H2AX foci evaluation was also used for characterization of RIBE both *in vitro* (51, 52) and *in vivo* (53). Sokolov et al. demonstrated that γ-H2AX co-localized with proteins involved in DNA damage response in bystander human fibroblast cultures (52). Here, we have demonstrated that BM-derived EVs from irradiated mice induced phosphorylation of the H2AX protein in EV-recipient bystander animals. The role of EV in mediating radiation-induced DNA damage in non-irradiated cells has not been reported yet. However, Dutta et al. showed that EVs isolated from the cell culture supernatant of human breast cancer cell lines were able to induce phosphorylation of key proteins (ataxia-teleangiectasia mutated (ATM), H2AX, Chk1, and p53) involved in DNA damage response in primary mammary epithelial cells *in vitro* by transmitting signals that led to ROS production and a consequential oxidative stress in the EV-recipient cells (54, 55). The role of EVs in inducing oxidative stress and mediating redox-regulated signaling processes in EV-recipient cells has been shown by several other recent reports as well (55, 56). Fontaine et al. proved the implicit role of EVs in this process, since the increased oxidative stress in the vascular wall of patients after coronary surgery disappeared if using EV-depleted plasma (56). It has been shown that EVs from preeclamptic women were directly taken up by endothelial cells leading to iNOS synthesis and activation of NFκB (57). Lee et al. found that hyperoxia-induced oxidative stress in lung epithelial cells led to increased EV production which in turn was taken up by macrophages leading to macrophage activation and increased production of NFκB-regulated pro-inflammatory molecules (44). It is known that ROS are mainly responsible for X-ray-induced DNA damage and activation of the DNA damage response pathways and the above publications prove that EVs are able to transmit oxidative stress in recipient cells. Thus, although we have not determined ROS levels in EV-recipient cells, it is logical to assume that the development of complex DNA damage consisting in increased IR-specific chromosomal aberrations and activation of the DNA damage response pathway in naïve mice receiving EVs from irradiated animals was mediated *via* redox-regulated signaling. This conclusion is supported by the fact that mice receiving EVs from non-irradiated mice showed background levels of DNA damage. Furthermore, as detailed later in this section, several pathways involved in DNA damage repair have been regulated by miRNA differentially expressed in EVs originating from the irradiated animals. While the above cited references point to a specific effect of EVs in recipient cells, the data published by Lee et al. raises the possibility of a systemic amplification and dissemination of the original EV-transmitted bystander signals by immune and inflammatory mediators released by activated immune cells (44). These data highlight the need for further research focusing on specific uptake of EVs by individual cellular subpopulations in the spleen and the subsequent cellular and molecular consequences.

Another interesting result was that both the level of γH2AX foci and the frequency of chromosomal aberrations were maximal when EVs were isolated from mice irradiated with 0.25 Gy. While we cannot explain this phenomenon, it harmonizes with other observed responses where the number of aberrations peaks at doses below 0.5 Gy (58–60). It was shown that RIBE are independent from the dose, instead the DNA repair capacity of the cell and amount of free radicals are more important factors (5). Most probably the explanation relies in the different macromolecular cargo of EVs released after low- and high-dose irradiation.

Next, we have studied phenotypical changes in the BM and spleen of the EV-recipient bystander mice by investigating changes in the pool, proliferation kinetics and activation status of various cellular subsets of the spleen and BM. It had been previously shown that BM stem and progenitor cells were very radiosensitive and that high-dose irradiation induced immediate damage in the various cellular subsets of the BM (61, 62). Our findings are partially in line with these reports, since we have detected strong reduction of the stem cell and lymphoid progenitor cell compartments after irradiation with 2 Gy but the myeloid progenitors and the megakaryocyte precursors did not change significantly. This might be explained by the fact that the manifestation of the radiation damage in these cells is delayed and the cytotoxic effect cannot be observed 24 h after irradiation. A very interesting observation in our study was that, in directly irradiated mice, stem cell numbers decreased to almost similar levels after low-dose irradiation (0.1 and 0.25 Gy), as after 2 Gy. It is unlikely that the strong reduction in stem cell numbers after low-dose irradiation is due to radiation-induced direct cell death, thus other mechanisms may be involved in this process. Previously, it was reported by Li et al. that low-dose irradiation induced a pronounced mobilization of BM stem cells to the periphery *via* a bystander mechanism, through increasing the systemic production of certain colony stimulating factors (63). This observation might explain the results obtained by us showing low-dose irradiation induced reduction of stem cells in the BM. Phenotypical changes in the BM cells of the EV-recipient mice were restricted to the stem cells only, where a moderate cell number reduction was detected after 0.25 and 2 Gy irradiations. Further studies are needed to elucidate the role of EVs in these bystander processes; however, a possible mechanism could be the one described above, where EV-mediated systemic bystander signals induce the mobilization of the stem cells into the periphery.

The increased radiosensitivity of the spleen is mainly due to its lymphocyte content, since lymphocytes are among the most radiosensitive cells in the body and even low radiation doses lead to significant lymphopenia. Formerly, we have reported strong differences in the radiosensitivity of the various lymphocyte subpopulations (64). In accordance with these results, here, we show that B and CD8^+^ T cells were more radiosensitive, while NK cells and DCs were more radiation resistant in the directly irradiated mice. Similar to our other formerly reported data (65), here we have found significantly increased apoptotic frequencies in the murine lymphocytes 24 h after irradiation. Radiation also inhibited the proliferative potential of all the investigated lymphocyte subpopulations in directly irradiated mice.

Regarding cell number changes, bystander responses in the spleen of EV-recipient mice resembled the direct radiation effects but had certain special characteristics, which indicate a different mechanism. These characteristics are the following: bystander responses were present only in certain splenocyte subpopulations (CD4^+^, CD8^+^ T and NK cells) and were absent in others (B cells and DCs). Bystander changes did not always follow the pattern of changes in the directly irradiated animals. For instance, EV derived from animals irradiated with 0.1 Gy induced statistically significant decrease in the CD4^+^ and CD8^+^ T cell pool and proliferation rate, NK cell proliferation rate in the same group of animals was increased, while these changes were absent in the directly irradiated animals. The most interesting was the way how splenic DC activation responded to radiation-induced direct and bystander stimuli. An increase was detected in the fraction of splenic DCs expressing TLR4 in the directly irradiated cells especially after irradiation with 2 Gy. This is in line with published data demonstrating that irradiation leads to increased release of danger signals, such as HMGB1, which interact with DCs *via* their TLR4 receptor (66–68). However, in bystander animals, the fraction of TLR4-expressing DCs decreased to half of the control level and changes were not influenced by radiation dose. These data indicate that EV-transmitted bystander signals inhibit or diminish DC response toward danger signals. Recent reviews have also identified TLRs as key molecules in radiation-induced systemic effects and inflammatory responses (3, 4) as well as one of the main pathways participating in radiation-induced systemic bystander effects.

We think that the above described phenotypical changes detected in EV-recipient mice support the idea that RIBE is not a passive transfer of radiation effects from directly irradiated cells to the bystander ones, but it is a rather selective process, involving complex signaling pathways, which influence multiple parameters in the recipient cells and the pattern of changes does not always reflect direct radiation effects. This assumes the presence of a panel of signaling molecules. Based on the above rationale EVs, which are active carriers of a multitude of signaling molecules (proteins, mRNAs, and miRNA), have a significant role in mediating RIBE.

MicroRNAs are evolutionarily conserved, small (~22 nucleotide long) non-coding RNAs, involved in transcriptional and posttranscriptional regulation of biological processes (69). Recently, it has been shown that EVs are rich sources of miRNAs, since, being packed in membrane-coated vesicles, they are more protected from RNAses than in a naked form (70, 71). The miRNA content of EVs does not necessarily reflect the miRNA of the cells that excrete them, since certain miRNAs are more abundant in EVs, indicating a specific packaging of miRNAs in EVs (72).

MicroRNAs were associated with tissue radiation response (73) and were potent inducers of RIBE (12, 74–76). The importance of miRNAs in cellular radiation response was demonstrated at a global level when Dicer and Drosha, the two key polymerases regulating miRNA biogenesis were knocked down in cells, which resulted in a reduction in the DNA damage response activation after IR (77) and in an increase in the radiosensitivity of the cells (78). Several publications reported that miRNAs were regulated by both low and high doses of IR in different tissues, including the hematopoietic system (79–81). Recent studies suggested that miRNAs carried by EVs were important mediators of radiation effects. Xu et al. showed that miRNAs could be transferred from irradiated cells to bystander cells through exosomes secreted in the cell culture medium and were able to induce RIBE (22). Al-Mayah et al. demonstrated that both cell supernatant and exosomes treated with RNAse lost their capacity to induce RIBE and genomic instability in MCF7 cells (20).

Since EVs are a rich source of miRNAs, able to transmit epigenetic signals from donor (in our case directly irradiated) cells to recipient (in our system bystander) cells and thus to modulate gene expression of recipient cells, we analyzed the miRNA cargo of BM-derived EVs originating from the directly irradiated animals. We found that the type of miRNAs was not different in the control and irradiated animals, it was rather the amount of individual miRNAs which was altered. This might be due to a radiation-induced difference in the expression of the miRNAs and/or to a radiation-induced selective packaging of miRNAs. The set of eight miRNAs which were differentially expressed in EVs after both low- and high-dose radiation seemed to be modulated dose dependently (Figure 8). Almost all eight miRNAs were found to modulate the radiation sensitivity of different tissues. miR-33 inhibited high-density lipoprotein-induced radiation sensitivity in breast cancer (82), and miR-199a-5p was found to sensitize breast cancer cells to irradiation (83). Several miRNAs were connected to DNA damage repair as well such as miR-33 and miR-375, which were shown to regulate DNA damage checkpoint through the p53 (82, 84) and miR-744-3p, which significantly delayed IR-induced DNA damage repair by directly targeting RAD23B in prostate cancer cells (85).

Several of the eight differentially expressed miRNAs were implicated in the regulation of certain immune processes. Thus, miR-152, which according to Wang et al. was upregulated by IR in certain human cell lines (86) controlled different cellular components of the innate immunity. Liu et al. showed that miR-152 negatively regulated DC maturation and activation by TLR4 agonists (such as LPS or HMGB1) (87). Increased miR-152 levels were associated with an increase in the killing activity of NK cells (88). Since in our study miR-152 levels increased in the EVs of both 0.1 Gy and 2 Gy irradiated mice, this might explain why the level of TLR4-expressing splenic DCs decreased in the bystander mice receiving irradiated EVs, as well as why the proliferation rate of NK cells increased in the same animals.

Recently, miR-33 has also been implicated in the regulation of innate immunity by repressing the ATP-binding cassette A1 and G1 proteins in macrophages (89, 90). One of the main roles of the ABCA1/G1 proteins is to inhibit the assembly and activation of TLR4 (91). This means that lower miR-33 levels could indirectly induce lower TLR4 levels, which was the case in our bystander animals.

We found 27 KEGG pathways predicted to be influenced by these eight differentially expressed miRNAs. Part of them is responsible for cellular radiation response and DNA repair (Hippo, Foxo, PI3K, Hedgehog, and TGFβ signaling pathways). Hippo pathway has been recently established as responsive to DNA damage, being activated by DNA strand breaks. It activates ATM and ATM- and RAD3-related (ATR) kinases, major regulators in DNA damage response. On the other hand, Hippo pathway can induce cell death in response to DNA damage (92). Foxo and PI3K pathways are also important in the ATM pathway activation and the maintenance of genome integrity in response to DNA damage (93, 94), while Hedgehog has a role in the DNA repair mechanisms (95). Three other pathways are strongly connected to the hematopoietic system. Interestingly, “Signaling pathways regulating pluripotency of stem cells” was one of the most significantly targeted pathways in our study, pointing to the functional changes we obtained in the hematopoietic stem cell populations. Wnt signaling pathway is a critical regulator of the balance of self-renewal and differentiation of the hematopoietic system, particularly of hematopoietic stem cells (96). Elements of Wnt-1 pathway can be found in different stages and sites of hematopoiesis. It is also an important pathway in splenic T-cell maturation (97), lymphoid progenitor cells, and different lymphoid subpopulations: it enhances CD8^+^ T cell production, regulatory CD4^+^ T cell survival, and B cell proliferation, as reviewed by Lento et al. (96). HTLV infections pathway incorporates parts of TGFβ-, T-cell receptor-, and Wnt signaling pathways. The endpoints of this pathway include inflammation, leukocyte migration, and proliferation; thereby it is also important in transmitting changes in hematopoietic system (98, 99). Our results are also in line with the findings of several recent reviews where the authors mapped the association of radiation with inflammatory and immune responses. In order to gather the most important biological molecules involved in RIBE, Nikitaki et al. using text and data mining created two lists of genes: genes implicated in bystander (closer to irradiated field) effects and systemic (at sites distant from the irradiated volume) effects and made a pathway enrichment analysis for each gene list. Among the top 10 pathways were chemokine, MAPK, and Jak–Stat signaling pathways as involved in bystander effects, and MAPK signaling, NK cell-mediated cytotoxicity and T cell receptor signaling pathways involved in systemic effects (5), with an excellent overlap with the pathways identified in our study as being affected by differentially expressed miRNAs from irradiated mice. Georgakilas et al. collected genes involved both in radiation response and in immune and/or inflammatory response and made a functional enrichment analysis, identifying several genes and pathways as immune and inflammatory response elements to radiation, among others TGFβ, WNT-, MAPK-, and insulin signaling (3), all of them being affected by miRNAs differentially expressed in the EVs from irradiated mice in our study.

Taking into consideration the potential role of the above-mentioned pathways in the induction of the EV-mediated systemic effects shown in our study, we constructed a hypothetical model based on these pathways. By selecting those elements from these pathways which were co-regulated by more than one differentially expressed miRNA, we pointed the potential genes which might be the effectors of the observed systemic changes mediated by EVs (Figure 10). Furthermore, when uploading and coupling these genes in FunCoup software, we found a set of enriched signaling pathways with all the members closely related to hematopoiesis, strongly reflecting the functional findings of our study: T cell signaling, B cell signaling, NK-mediated cytotoxicity, chemokine signaling, Fc epsilon signaling, insulin signaling, Jak–Stat signaling, and Wnt signaling pathways (Table 1). The other three enriched pathways, TGFβ, ErbB, and MAPK pathways are broad signal transduction pathways governing cell proliferation and survival. We think that the genes and pathways from this model could be important players in the mechanisms of the observed bystander effects and their individual role in this process worth being further elucidated.

In conclusion, we have established an *in vivo* model system suitable to study the role of EVs in mediating radiation effects in EV-recipient mice. We demonstrated that BM-derived EVs originating from irradiated mice activated DNA damage response in the spleen of the EV-recipient bystander animals and induced quantitative and phenotypical changes in the stem and progenitor cell compartment of the BM and in the different splenocyte subpopulations. These systemic effects were present at low radiation doses as well and they did not show any correlation with the dose in most of the cases. Furthermore, the pattern of changes was often different from that observed in the directly irradiated animals, indicating that the mechanisms responsible for these effects were also different. Given the rich miRNA content of EVs and the fact that miRNAs are considered as potential mediators of RIBE, we performed a miRNA analysis of the EVs and identified eight miRNAs in the BM-derived EVs of irradiated animals, which were differentially expressed in both the low- and high-dose-irradiated samples. A thorough database and network analysis of these miRNAs showed their potential involvement in pathways regulating DNA damage response, hematopoiesis, and different immune functions. Some of these miRNAs were experimentally validated by others to modulate innate immunity. Based on these findings, we have constructed a hypothetical network of miRNAs, their target mRNAs, and pathways which might be the most relevant in our system for mediating systemic RIBE. While the role of these individual miRNAs has to be verified experimentally, we think we could clearly demonstrate that EVs are mediators of systemic RIBE most probably *via* their miRNA cargo.

## Author Contributions

TS did miRNA analysis, γ-H2AX assay by fluorescent microscopy. DK performed and evaluated bone marrow phenotyping, and contributed in writing the manuscript. EB did spleen immune phenotyping, apoptosis analysis, and γ-H2AX assay by flow cytometry and revised manuscript. ABenedek, EK, DK, and EB performed animal treatments and isolated EVs. ABalogh performed EV characterization, participated in microRNA analysis, and revised the manuscript, LN performed EV size distribution analysis, and EP did Western blotting and revised the manuscript. SB, DB, and MK did chromosomal analysis, electron microscopy, and revised the manuscript. KL designed the experiments. TS, GS, and KL wrote the manuscript.

## Conflict of Interest Statement

The authors declare that the research was conducted in the absence of any commercial or financial relationships that could be construed as a potential conflict of interest.

## Footnotes

^1^www.graphpad.com.

^2^www.storedb.org.

## Abbreviations

AchE, acetylcholinesterase; BM, bone marrow; BSA, bovine serum albumin; Cq, quantification cycles; DC, dendritic cell; DSB, double-strand break; EV, extracellular vesicles; FOXO, forkhead box O; GO, Gene Ontology; HMGB1, high-mobility group box 1 protein; HTLV, human T cell lymphotrophic virus; IR, ionizing radiation; KEGG, Kyoto Encyclopedia of Genes and Genomes; LPS, lipopolysaccharide; MV, microvesicles; NFκB, nuclear factor kappa b; NK, natural killer cell; PBS, phosphate-buffered saline; PCR, polymerase chain reaction; PFA, paraformaldehyde; PI3K, phosphoinositide 3-kinase; RIBE, radiation-induced bystander effects; RT, room temperature; TGFβ, transforming growth factor beta; TLR, toll-like receptor.
