# Supplementary material for: Extracellular Vesicles Mediate Radiation-Induced Systemic Bystander Signals in the Bone Marrow and Spleen
Source: Front Immunol. 2017 Mar 27;8:347. doi: 10.3389/fimmu.2017.00347 (PMC5366932; doi:10.3389/fimmu.2017.00347)
Supplement: Supplementary file 1 [file Table_1.DOCX]

**Supplementary Table 1. Differentially expressed miRNAs in 0.1 Gy vs 0 Gy groups.**

Mean FC refers to mean fold change in the expression of the corresponding miRNA

| **miR 0.1Gy vs 0 Gy** | **Mean FC** | **P value** |
| --- | --- | --- |
| mmu-miR-1941-3p | -1.97 | 0.011 |
| mmu-miR-33-3p | -1.65 | 0.019 |
| mmu-miR-200c-5p | -1.58 | 0.022 |
| mmu-miR-127-5p | -1.54 | 0.038 |
| mmu-miR-615-3p | -1.51 | 0.032 |
| mmu-miR-574-3p | -1.42 | 0.039 |
| mmu-miR-376b-5p | -1.42 | 0.008 |
| mmu-miR-379-3p | -1.40 | 0.001 |
| mmu-miR-140-3p | -1.28 | 0.006 |
| mmu-miR-677-5p | -1.22 | 0.039 |
| mmu-miR-744-3p | -1.17 | 0.025 |
| mmu-miR-211-5p | -1.14 | 0.023 |
| mmu-miR-669o-5p | -1.14 | 0.015 |
| mmu-miR-125b-5p | -1.08 | 0.041 |
| mmu-miR-125a-5p | 1.11 | 0.037 |
| mmu-miR-152-3p | 1.13 | 0.016 |
| mmu-miR-10a-5p | 1.16 | 0.042 |
| mmu-miR-199a-5p | 1.23 | 0.016 |
| mmu-miR-376a-3p | 1.35 | 0.012 |
| mmu-miR-375-3p | 1.74 | 0.138 |
