# Supplementary material for: Extracellular Vesicles Mediate Radiation-Induced Systemic Bystander Signals in the Bone Marrow and Spleen
Source: Front Immunol. 2017 Mar 27;8:347. doi: 10.3389/fimmu.2017.00347 (PMC5366932; doi:10.3389/fimmu.2017.00347)
Supplement: Supplementary file 2 [file Table_2.DOCX]

**Supplementary Table 2. Differentially expressed miRNAs in 2 Gy vs 0 Gy groups.**

Mean FC refers to mean fold change in the expression of the corresponding miRNA

| **miR 2Gy vs 0 Gy** | **Mean FC** | **p value** |
| --- | --- | --- |
| mmu-miR-592-5p | -14.73 | <0.001 |
| mmu-miR-192-3p | -6.15 | 0.002 |
| mmu-miR-150-5p | -5.27 | 0.007 |
| mmu-miR-2136 | -5.14 | 0.018 |
| mmu-miR-466f-3p | -4.74 | 0.000 |
| mmu-miR-1902 | -4.72 | 0.015 |
| mmu-miR-763 | -4.60 | 0.002 |
| mmu-miR-1929-5p | -4.48 | 0.004 |
| mmu-miR-1839-3p | -4.38 | 0.007 |
| mmu-miR-467d-3p | -4.35 | 0.001 |
| mmu-miR-33-3p | -4.24 | 0.006 |
| mmu-miR-467f | -3.90 | 0.018 |
| mmu-miR-374b-5p | -3.89 | 0.006 |
| mmu-miR-468-3p | -3.89 | 0.005 |
| mmu-miR-669a-5p | -3.39 | 0.003 |
| mmu-miR-93-5p | -3.28 | 0.005 |
| mmu-miR-491-5p | -3.27 | 0.001 |
| mmu-miR-299a-5p | -3.27 | 0.037 |
| mmu-miR-182-5p | -3.20 | 0.014 |
| mmu-miR-1933-5p | -3.04 | 0.040 |
| mmu-miR-200c-3p | -2.97 | 0.014 |
| mmu-miR-872-5p | -2.95 | 0.002 |
| mmu-miR-30c-5p | -2.79 | 0.006 |
| mmu-miR-421-3p | -2.67 | 0.013 |
| mmu-miR-192-5p | -2.62 | 0.011 |
| mmu-miR-26a-1-3p | -2.62 | 0.042 |
| mmu-miR-93-3p | -2.61 | 0.009 |
| mmu-miR-25-3p | -2.60 | 0.009 |
| mmu-miR-709 | -2.58 | 0.004 |
| mmu-miR-340-3p | -2.55 | 0.029 |
| mmu-miR-31-3p | -2.51 | 0.045 |
| mmu-miR-130b-5p | -2.42 | 0.000 |
| mmu-miR-669o-5p | -2.40 | 0.004 |
| mmu-miR-29a-5p | -2.38 | 0.001 |
| mmu-miR-1949 | -2.37 | 0.001 |
| mmu-miR-219a-1-3p | -2.31 | 0.003 |
| mmu-miR-489-3p | -2.31 | 0.020 |
| mmu-miR-3107-5p | -2.29 | 0.021 |
| mmu-miR-466d-3p | -2.28 | 0.028 |
| mmu-miR-92a-3p | -2.24 | <0.0001 |
| mmu-miR-449a-5p | -2.22 | 0.019 |
| mmu-miR-1966-5p | -2.19 | 0.048 |
| mmu-miR-17-5p | -2.18 | 0.042 |
| mmu-miR-1191 | -2.13 | 0.040 |
| mmu-miR-185-5p | -2.07 | 0.014 |
| mmu-miR-7a-1-3p | -2.06 | 0.011 |
| mmu-miR-363-3p | -2.01 | 0.006 |
| mmu-let-7g-5p | -2.01 | 0.001 |
| mmu-miR-181a-1-3p | -2.00 | 0.014 |
| mmu-miR-18a-3p | -1.99 | 0.006 |
| mmu-miR-200c-5p | -1.98 | 0.016 |
| mmu-miR-669l-5p | -1.97 | 0.017 |
| mmu-miR-191-5p | -1.94 | 0.011 |
| mmu-miR-1930-5p | -1.94 | 0.013 |
| mmu-miR-465a-3p | -1.90 | 0.025 |
| mmu-let-7d-5p | -1.90 | 0.047 |
| mmu-miR-1934-5p | -1.89 | 0.021 |
| mmu-miR-20b-3p | -1.87 | 0.018 |
| mmu-miR-30a-3p | -1.83 | 0.036 |
| mmu-miR-296-5p | -1.81 | 0.050 |
| mmu-miR-9-5p | -1.75 | 0.046 |
| mmu-miR-322-3p | -1.75 | 0.033 |
| mmu-miR-1198-3p | -1.74 | 0.026 |
| mmu-miR-184-3p | -1.74 | 0.023 |
| mmu-miR-17-3p | -1.69 | 0.009 |
| mmu-miR-500-3p | -1.67 | 0.023 |
| mmu-miR-20a-5p | -1.64 | 0.003 |
| mmu-miR-331-5p | -1.55 | 0.011 |
| mmu-miR-138-5p | -1.54 | 0.022 |
| mmu-miR-551b-3p | -1.49 | 0.050 |
| mmu-miR-744-3p | -1.47 | 0.016 |
| mmu-miR-146a-5p | -1.45 | 0.020 |
| mmu-miR-193b-5p | -1.42 | 0.029 |
| mmu-miR-140-3p | -1.41 | 0.014 |
| mmu-miR-466h-5p | -1.41 | 0.037 |
| mmu-miR-24-3p | -1.32 | 0.040 |
| mmu-miR-221-3p | 1.93 | 0.043 |
| mmu-miR-199a-5p | 2.06 | 0.009 |
| mmu-miR-152-3p | 2.06 | 0.028 |
| mmu-miR-676-5p | 2.10 | 0.045 |
| mmu-miR-145a-5p | 2.19 | 0.025 |
| mmu-miR-203-5p | 2.20 | 0.035 |
| mmu-miR-147-3p | 2.31 | 0.032 |
| mmu-miR-199b-5p | 2.34 | 0.041 |
| mmu-miR-22-5p | 2.60 | 0.049 |
| mmu-miR-196b-3p | 3.53 | 0.029 |
| mmu-miR-224-5p | 3.66 | 0.030 |
| mmu-miR-340-5p | 4.10 | 0.025 |
| mmu-miR-145a-3p | 4.47 | 0.028 |
| mmu-miR-375-3p | 2.17 | 0.071 |
