# Supplementary material for: Extracellular Vesicles Mediate Radiation-Induced Systemic Bystander Signals in the Bone Marrow and Spleen
Source: Front Immunol. 2017 Mar 27;8:347. doi: 10.3389/fimmu.2017.00347 (PMC5366932; doi:10.3389/fimmu.2017.00347)
Supplement: Supplementary file 3 [file Table_3.DOCX]

**Supplementary Table 3. Top KEGG pathways predicted to be targeted by the miRNAs differentially expressed in 0.1 Gy samples**

Number of genes refer to the number of mRNAs involved in the corresponding pathway, number of miRNAs refer to the number of miRNAs supposed to regulate the corresponding pathway.

| **KEGG pathway** | **p-value** | **nr. genes** | **nr. miRNAs** |
| --- | --- | --- | --- |
| Hippo signalling pathway | 2.03E-07 | 46 | 17 |
| Proteoglycans in cancer | 2.03E-07 | 52 | 16 |
| Signalling pathways regulating pluripotency of stem cells | 0.000266 | 35 | 17 |
| Adrenergic signalling in cardiomyocytes | 0.00055 | 37 | 18 |
| MAPK signalling pathway | 0.001103 | 57 | 16 |
| Wnt signalling pathway | 0.001103 | 34 | 17 |
| Lysine degradation | 0.003582 | 11 | 12 |
| mRNA surveillance pathway | 0.003582 | 27 | 13 |
| Hypertrophic cardiomyopathy (HCM) | 0.004084 | 25 | 16 |
| Melanogenesis | 0.004084 | 27 | 17 |
| Endocytosis | 0.004259 | 51 | 13 |
| Oxytocin signalling pathway | 0.004259 | 38 | 16 |
| Neurotrophin signalling pathway | 0.008092 | 30 | 13 |
| AMPK signalling pathway | 0.009205 | 30 | 14 |
| Hedgehog signalling pathway | 0.009205 | 16 | 12 |
| Estrogen signalling pathway | 0.011321 | 19 | 12 |
| Other glycan degradation | 0.011321 | 2 | 3 |
| Basal cell carcinoma | 0.011659 | 17 | 14 |
| Dilated cardiomyopathy | 0.011659 | 24 | 17 |
| Long-term potentiation | 0.011659 | 19 | 14 |
| FoxO signalling pathway | 0.013529 | 32 | 14 |
| Glycosphingolipid biosynthesis - lacto and neolacto series | 0.013529 | 4 | 8 |
| PI3K-Akt signalling pathway | 0.014054 | 66 | 16 |
| Amphetamine addiction | 0.014434 | 17 | 13 |
| Axon guidance | 0.014552 | 32 | 13 |
| Endocrine and other factor-regulated calcium reabsorption | 0.015145 | 15 | 11 |
| Thyroid hormone signalling pathway | 0.019841 | 22 | 14 |
| Insulin signalling pathway | 0.035023 | 31 | 15 |
| Ubiquitin mediated proteolysis | 0.035023 | 32 | 13 |
| HTLV-I infection | 0.039956 | 51 | 17 |
| Cell adhesion molecules (CAMs) | 0.041343 | 27 | 13 |
| Protein processing in endoplasmic reticulum | 0.041343 | 33 | 14 |
| TGF-beta signalling pathway | 0.041343 | 21 | 14 |
