# Supplementary material for: Extracellular Vesicles Mediate Radiation-Induced Systemic Bystander Signals in the Bone Marrow and Spleen
Source: Front Immunol. 2017 Mar 27;8:347. doi: 10.3389/fimmu.2017.00347 (PMC5366932; doi:10.3389/fimmu.2017.00347)
Supplement: Supplementary file 4 [file Table_4.DOCX]

**Supplementary Table 4. Top KEGG pathways predicted to be targeted by the miRNAs differentially expressed in 2 Gy samples**

Number of genes refer to the number of mRNAs involved in the corresponding pathway, number of miRNAs refer to the number of miRNAs supposed to regulate the corresponding pathway.

| **KEGG pathway** | **p-value** | **nr. genes** | **nr. miRNAs** |
| --- | --- | --- | --- |
| Hippo signalling pathway | 3.13E-12 | 100 | 63 |
| Axon guidance | 1.49E-10 | 91 | 71 |
| Proteoglycans in cancer | 2.51E-10 | 124 | 74 |
| Mucin type O-Glycan biosynthesis | 9.47E-10 | 17 | 28 |
| ECM-receptor interaction | 1.53E-09 | 44 | 46 |
| Prion diseases | 1.23E-08 | 18 | 28 |
| Phosphatidylinositol signalling system | 1.20E-07 | 55 | 57 |
| Adherens junction | 1.27E-07 | 52 | 54 |
| GABAergic synapse | 3.04E-07 | 48 | 48 |
| Pathways in cancer | 5.63E-07 | 205 | 72 |
| Thyroid hormone signalling pathway | 5.63E-07 | 71 | 62 |
| MAPK signalling pathway | 5.66E-07 | 142 | 72 |
| Regulation of actin cytoskeleton | 5.66E-07 | 122 | 66 |
| FoxO signalling pathway | 1.58E-06 | 84 | 68 |
| cAMP signalling pathway | 1.99E-06 | 115 | 71 |
| Wnt signalling pathway | 3.24E-06 | 85 | 67 |
| Focal adhesion | 1.02E-05 | 118 | 67 |
| Inositol phosphate metabolism | 1.14E-05 | 40 | 49 |
| Neurotrophin signalling pathway | 4.97E-05 | 73 | 62 |
| Signalling pathways regulating pluripotency of stem cells | 4.97E-05 | 78 | 65 |
| TGF-beta signalling pathway | 4.97E-05 | 50 | 56 |
| Arrhythmogenic right ventricular cardiomyopathy (ARVC) | 7.09E-05 | 44 | 53 |
| Choline metabolism in cancer | 7.09E-05 | 62 | 63 |
| ErbB signalling pathway | 0.000104 | 50 | 60 |
| Amphetamine addiction | 0.000147 | 41 | 60 |
| Nicotine addiction | 0.000147 | 24 | 37 |
| Renal cell carcinoma | 0.000221 | 41 | 56 |
| PI3K-Akt signalling pathway | 0.000253 | 176 | 74 |
| T cell receptor signalling pathway | 0.000688 | 61 | 63 |
| Rap1 signalling pathway | 0.000837 | 112 | 67 |
| Oxytocin signalling pathway | 0.000981 | 87 | 71 |
| mTOR signalling pathway | 0.001004 | 39 | 60 |
| Colorectal cancer | 0.001059 | 39 | 51 |
| Osteoclast differentiation | 0.001291 | 70 | 60 |
| Hepatitis B | 0.001682 | 70 | 62 |
| Protein processing in endoplasmic reticulum | 0.001694 | 90 | 61 |
| Endocytosis | 0.00189 | 114 | 68 |
| Notch signalling pathway | 0.00189 | 32 | 45 |
| Ras signalling pathway | 0.002167 | 112 | 70 |
| AMPK signalling pathway | 0.002284 | 68 | 66 |
| Thyroid hormone synthesis | 0.004249 | 36 | 47 |
| Long-term potentiation | 0.004891 | 40 | 65 |
| Insulin signalling pathway | 0.005203 | 76 | 69 |
| Prostate cancer | 0.005203 | 50 | 59 |
| Melanogenesis | 0.005374 | 55 | 63 |
| Bacterial invasion of epithelial cells | 0.006507 | 44 | 46 |
| Type II diabetes mellitus | 0.007042 | 30 | 44 |
| Fc gamma R-mediated phagocytosis | 0.007129 | 49 | 57 |
| Pancreatic cancer | 0.007675 | 38 | 52 |
| Calcium signalling pathway | 0.007907 | 92 | 65 |
| Acute myeloid leukemia | 0.009524 | 33 | 49 |
| Glutamatergic synapse | 0.010422 | 57 | 53 |
| N-Glycan biosynthesis | 0.011 | 23 | 27 |
| Dilated cardiomyopathy | 0.011621 | 48 | 63 |
| Inflammatory bowel disease (IBD) | 0.012792 | 28 | 39 |
| mRNA surveillance pathway | 0.015591 | 51 | 61 |
| Amoebiasis | 0.016509 | 55 | 52 |
| Hypertrophic cardiomyopathy (HCM) | 0.016509 | 45 | 62 |
| Basal cell carcinoma | 0.019579 | 33 | 44 |
| Insulin secretion | 0.019686 | 47 | 58 |
| Adrenergic signalling in cardiomyocytes | 0.020686 | 78 | 65 |
| SNARE interactions in vesicular transport | 0.020709 | 19 | 28 |
| Glycosaminoglycan biosynthesis - keratan sulfate | 0.021038 | 10 | 17 |
| Transcriptional misregulation in cancer | 0.023132 | 90 | 71 |
| Glycosaminoglycan biosynthesis - heparan sulfate / heparin | 0.027323 | 11 | 22 |
| Circadian rhythm | 0.031973 | 18 | 45 |
| HTLV-I infection | 0.032001 | 128 | 72 |
| Salivary secretion | 0.032746 | 41 | 47 |
| Lipoic acid metabolism | 0.040331 | 3 | 3 |
| Long-term depression | 0.043738 | 30 | 53 |
| Dorso-ventral axis formation | 0.045721 | 16 | 47 |
| Fc epsilon RI signalling pathway | 0.045721 | 37 | 57 |
| Glioma | 0.045721 | 32 | 59 |
| HIF-1 signalling pathway | 0.045721 | 56 | 55 |
| TNF signalling pathway | 0.045721 | 54 | 53 |
