# Supplementary material for: Extracellular Vesicles Mediate Radiation-Induced Systemic Bystander Signals in the Bone Marrow and Spleen
Source: Front Immunol. 2017 Mar 27;8:347. doi: 10.3389/fimmu.2017.00347 (PMC5366932; doi:10.3389/fimmu.2017.00347)
Supplement: Supplementary file 5 [file Table_5.DOCX]

**Supplementary Table 5. Gene Ontology categories predicted to be altered by miRNAs differentially expressed in both 0.1 and 2 Gy samples**

Number of genes refer to the number of mRNAs involved in the corresponding pathway, number of miRNAs refer to the number of miRNAs supposed to regulate the corresponding pathway.

| **GO biological process** | **p value** | **nr. genes** | **nr. miRNAs** |
| --- | --- | --- | --- |
| anatomical structure development | 6.98e-78 | 407 | 8 |
| cell differentiation | 5.41e-44 | 303 | 8 |
| embryo development | 1.33e-34 | 136 | 8 |
| anatomical structure formation involved in morphogenesis | 1.71e-21 | 109 | 8 |
| cell morphogenesis | 2.77e-18 | 95 | 8 |
| cellular nitrogen compound metabolic process | 4.25e-10 | 369 | 8 |
| biosynthetic process | 5.56e-09 | 325 | 8 |
| chromosome organization | 6.18e-09 | 64 | 7 |
| cellular protein modification process | 6.18e-09 | 205 | 8 |
| cell motility | 1.97e-06 | 68 | 8 |
| growth | 8.20e-06 | 52 | 7 |
| developmental maturation | 0.00017 | 22 | 7 |
| positive regulation of transcription | 0.0245 | 92 | 8 |
| cell death | 0.0271 | 78 | 7 |
| negative regulation of translation involved in gene silencing by miRNA | 0.0494 | 6 | 5 |
| **GO cellular component** | **p value** | **nr. genes** | **nr. miRNAs** |
| nuclear chromosome | 0.00593 | 26 | 6 |
| cytoplasmic stress granule | 0.0177 | 12 | 7 |
| cytoplasmic membrane-bounded vesicle | 0.0359 | 48 | 8 |
