# Supplementary material for: Extracellular Vesicles Mediate Radiation-Induced Systemic Bystander Signals in the Bone Marrow and Spleen
Source: Front Immunol. 2017 Mar 27;8:347. doi: 10.3389/fimmu.2017.00347 (PMC5366932; doi:10.3389/fimmu.2017.00347)
Supplement: Supplementary file 6 [file Table_6.DOCX]

**Supplementary Table 6. Significantly altered pathways based on KEGG pathway analysis in both 0.1 Gy and 2 Gy.** Number of genes refer to the number of mRNAs involved in the corresponding pathway, number of miRNAs refer to the number of miRNAs supposed to regulate the corresponding pathway.

| **KEGG pathway** | **p-value** | **nr. genes** | **nr. miRNAs** |
| --- | --- | --- | --- |
| Hippo signalling pathway | 1.94E-08 | 31 | 7 |
| FoxO signalling pathway | 2.61E-05 | 27 | 7 |
| Proteoglycans in cancer | 2.61E-05 | 30 | 7 |
| Signalling pathways regulating pluripotency of stem cells | 0.000141 | 22 | 7 |
| PI3K-Akt signalling pathway | 0.000315 | 45 | 7 |
| Wnt signalling pathway | 0.000664 | 19 | 8 |
| Hypertrophic cardiomyopathy (HCM) | 0.000824 | 18 | 7 |
| AMPK signalling pathway | 0.001312 | 20 | 7 |
| Melanogenesis | 0.003821 | 17 | 8 |
| Dilated cardiomyopathy | 0.004174 | 17 | 8 |
| Insulin signalling pathway | 0.00639 | 22 | 7 |
| Adrenergic signalling in cardiomyocytes | 0.008925 | 20 | 8 |
| Neurotrophin signalling pathway | 0.011023 | 20 | 6 |
| MAPK signalling pathway | 0.016549 | 32 | 7 |
| HTLV-I infection | 0.021008 | 30 | 8 |
| Hedgehog signalling pathway | 0.025913 | 10 | 5 |
| Pantothenate and CoA biosynthesis | 0.025913 | 3 | 2 |
| TGF-beta signalling pathway | 0.027431 | 13 | 7 |
| Lysine degradation | 0.031655 | 6 | 7 |
| GABAergic synapse | 0.031964 | 9 | 7 |
| Renal cell carcinoma | 0.039774 | 11 | 5 |
| Basal cell carcinoma | 0.043944 | 10 | 6 |
| D-Arginine and D-ornithine metabolism | 0.043944 | 1 | 2 |
| Glycosphingolipid biosynthesis - lacto and neolacto series | 0.043944 | 3 | 3 |
| Nicotine addiction | 0.043944 | 5 | 5 |
| Pathways in cancer | 0.043944 | 40 | 8 |
| Protein processing in endoplasmic reticulum | 0.043944 | 20 | 7 |
