# Supplementary figures and images for: Extracellular Vesicles Mediate Radiation-Induced Systemic Bystander Signals in the Bone Marrow and Spleen
Source: Front Immunol. 2017 Mar 27;8:347. doi: 10.3389/fimmu.2017.00347 (PMC5366932; doi:10.3389/fimmu.2017.00347)

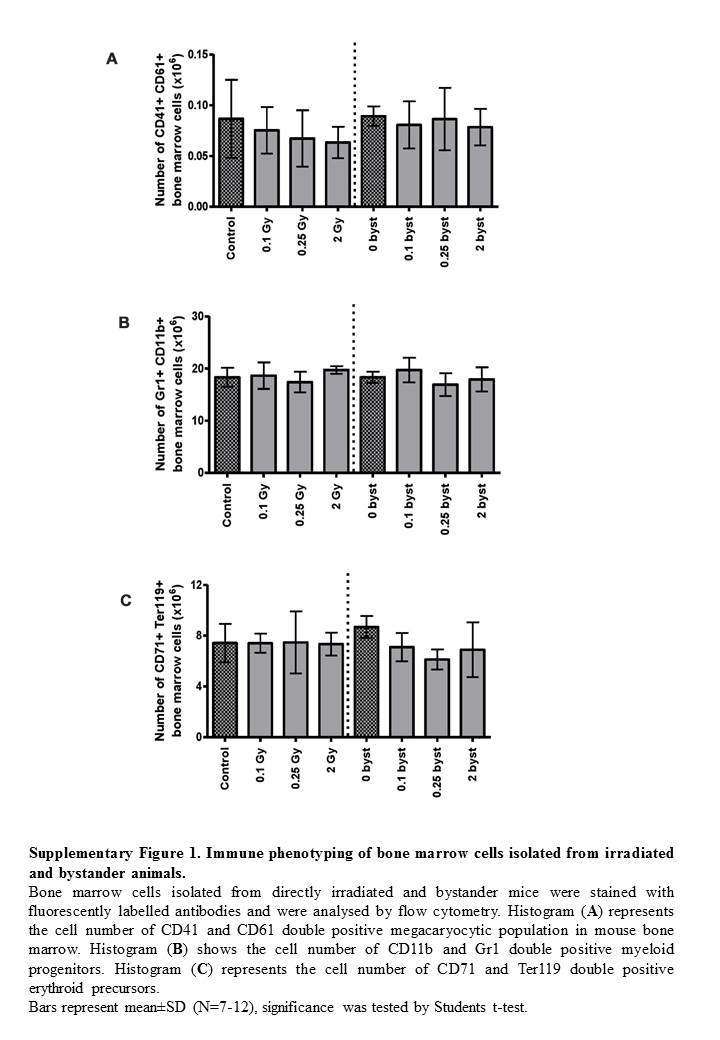

Supplement: Supplementary file 7 [file Image_1.JPEG]

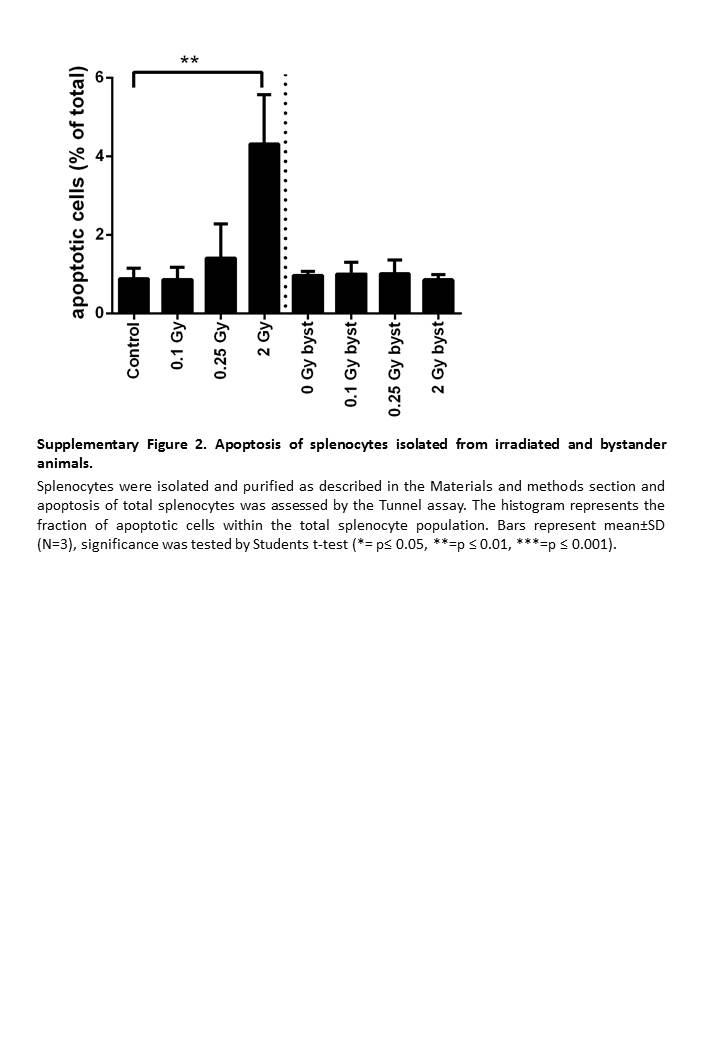

Supplement: Supplementary file 8 [file Image_2.JPEG]
